# Policies in Canada fail to address disparities in access to person-centred osteoarthritis care: a content analysis

**DOI:** 10.1186/s12913-024-10966-5

**Published:** 2024-04-25

**Authors:** Angelina Abbaticchio, Madeline Theodorlis, Deborah Marshall, Crystal MacKay, Cornelia M. Borkhoff, Glen Stewart Hazlewood, Marisa Battistella, Aisha Lofters, Vandana Ahluwalia, Anna R. Gagliardi

**Affiliations:** 1 Toronto General Hospital Research Institute, University Health Network, 200 Elizabeth Street, Toronto, M5G2C4 Canada; 2 University of Calgary, Calgary, Canada; 3 West Park Healthcare Centre, York, Canada; 4 Institute of Health Policy, Management and Evaluation, Dalla Lana School of Public Health, University of Toronto, Toronto, Canada; 5 Department of Family and Community Medicine, University of Toronto, Toronto, Canada; 6 William Osler Health System, Brampton, Canada

**Keywords:** Osteoarthritis, Patient-centred care, Healthcare equity, Women’s health, Equity-seeking groups, Policies, Content analysis

## Abstract

**Background:**

Women are disproportionately impacted by osteoarthritis (OA) but less likely than men to access OA care, particularly racialized women. One way to reduce inequities is through policies that can influence healthcare services. We examined how OA-relevant policies in Canada address equitable, person-centred OA care for women.

**Methods:**

We used content analysis to extract data from English-language OA-relevant documents referred to as policies or other synonymous terms published in 2000 or later identified by searching governmental and other web sites. We used summary statistics to describe policy characteristics, person-centred care using McCormack’s six-domain framework, and mention of OA prevalence, barriers and strategies to improve equitable access to OA care among women.

**Results:**

We included 14 policies developed from 2004 to 2021. None comprehensively addressed all person-centred care domains, and few addressed individual domains: enable self-management (50%), share decisions (43%), exchange information (29%), respond to emotions (14%), foster a healing relationship (0%) and manage uncertainty (0%). Even when mentioned, content offered little guidance for how to achieve person-centred OA care. Few policies acknowledged greater prevalence of OA among women (36%), older (29%) or Indigenous persons (29%) and those of lower socioeconomic status (14%); or barriers to OA care among those of lower socioeconomic status (50%), in rural areas (43%), of older age (37%) or ethno-cultural groups (21%), or women (21%). Four (29%) policies recommended strategies for improving access to OA care at the patient (self-management education material in different languages and tailored to cultural norms), clinician (healthcare professional education) and system level (evaluate OA service equity, engage lay health leaders in delivering self-management programs, and offer self-management programs in a variety of formats). Five (36%) policies recommended research on how to improve OA care for equity-seeking groups.

**Conclusions:**

Canadian OA-relevant policies lack guidance to overcome disparities in access to person-centred OA care for equity-seeking groups including women. This study identified several ways to strengthen policies. Ongoing research must identify the needs and preferences of equity-seeking persons with OA, and evaluate the impact of various models of service delivery, knowledge needed to influence OA-relevant policy.

**Supplementary Information:**

The online version contains supplementary material available at 10.1186/s12913-024-10966-5.

## Background

Osteoarthritis (OA) is defined as a primarily degenerative, sometimes inflammatory disease characterised by stiffness, inflammation, and physical and psychological impairments [1]. The Global Burden of Disease Study reported that prevalent cases of OA increased globally by greater than 113% over three decades, more than doubling from 247.51 million cases in 1990 to 527.81 million cases in 2019 [2]. OA prevalence is expected to increase, particularly for knee and hip joints [2]. OA can lead to poor quality of life, depression, diabetes, and heart disease; thus, early diagnosis and management are critical [3]. While guidelines vary, initial management (often referred to as first-line) for hand, hip and knee OA typically includes physical activity, pharmacologic and non-pharmacologic pain control, and self-management programs [4]. Subsequent or second-line therapy may include surgery or joint replacement [4].

Compared to men, OA is more prevalent and severe among women [2]. Women also experience a greater number of OA-related comorbid conditions compared to males [5]. However, women are less likely than men to receive early diagnosis and management of OA, and this is particularly true among racialized or immigrant women [6–10], many of whom may refrain from seeking care due to poor healthcare experiences [11]. Furthermore, many racialized or immigrant women have low rates of physical activity, an important first-line strategy to mitigate OA, due to numerous gendered, cultural and socioeconomic factors [12]. Hence, efforts are needed to improve access to and quality of OA care for diverse women.

A 2023 scoping review identified only 11 studies published after 2009 on interventions to reduce inequities in OA care among equity-seeking groups, of which only 2 focused soley on women [13]. Interventions largely consisted of OA self-management education, often delivered in community settings, which improved patient knowledge-based, behavioural and clinical outcomes [13]. Despite these promising findings, no interventions addressed other factors that contribute to inequitable OA care for diverse women. This is a notable gap because considerable research shows that barriers of OA care exist not only at the patient level, but also at the clinician (e.g. OA not considered serious, lack of time) and healthcare system levels (cost of therapies not covered, service availability) [14, 15]. It appears that complex, interacting, multi-level determinants may influence access to and quality of OA care. Thus, self-management education alone is not likely to greatly reduce inequities experienced by diverse women. Other research generated insight on what constitutes person-centred OA care. Consultations with 26 patients with OA and 147 healthcare professionals of 18 disciplines in 31 countries generated 70 quality indicators of person-centred OA care (e.g. identify the financial burden of treatment and patient preferences when planning care) [16]. Systematic review and expert consensus were used to generate 56 quality indicators of person-centred OA care [17]. Another systematic review and engagement of patients with OA resulted in 15 quality indicators of person-centred OA care [18]. While important to set standards, these initiatives did not identify concrete strategies needed to achieve these person-centred quality indicators, which may require healthcare system reforms [19].

A review of 24 systematic reviews including a total of 128 primary studies spanning eight public health domains (e.g. tobacco, food and nutrition, control of infectious diseases, screening) revealed that policies were more beneficial for reducing or preventing health inequities than educational campaigns, underscoring the important role of healthcare policies in shaping the organization and delivery of services [20]. “Policies” refers to documents, possibly labelled as policy, decision, plan, framework, strategy or synonymous term, that are generated by government or governmental agencies to guide the planning, funding, organization, delivery or improvement of healthcare programs or services [21]. Given the critical role of policies in promoting equitable access to high-quality care [20], perhaps in combination with other strategies, the overall aim of this study was to examine the content of policies for strategies that improve OA care for all. This is germane in Canada, where a large proportion of the population is comprised of immigrants [22], and where research showed that women experienced disparities in access to and quality of OA care [6]. The specific purpose of this study was to describe whether and how policies developed by Canadian governmental and other organizations (e.g. inter-sectoral consortia, advocacy groups) recognize and address equitable, person-centred OA care for diverse women. This knowledge could provide direction for strengthening policies in Canada, and possibly elsewhere, so as to ultimately improve OA care.

## Methods

### Approach

We used content analysis of policy documents to identify details related to strategies that support equitable, person-centred OA care, an approach that is commonly used to describe explicit information in any form of communication [23, 24]. Our approach was manifest, which refers to extracting and reporting explicit content. This approach involves both deductive and summative analysis to first organize content into categories (deductive), and then counting and comparing categories across policies (summative) [23, 24]. We did not require research ethics board approval because documents were publicly available. To enhance rigour, multiple team members (AA woman graduate student, MT woman research associate, ARG woman principal investigator) independently analyzed, then compared data to resolve discrepancies through discussion. Data were reviewed by the larger research team that included a 13-member advisory group of diverse women with lived experience of OA (ages 53 to 84; 10 to 40 + years with OA; OA of the back, hands, hips, knees, neck and sholders; 2 Ugandan, 2 Chinese, 1 Filipino, 1 Indigenous and 7 White/European), healthcare professionals (family physician, rheumatologists, physiotherapist, pharmacist) and health services researchers with expertise in the topics of OA, person-centred care, equity and women’s health. These differing characteristics and roles, and interaction among members of the research team throughout the study, contributed to a balance of perspectives in interpreting data.

### Eligibility

Additional File S1 describes detailed eligibility criteria, informed by prior research on OA disparities among women [13] and what constitutes person-centred care for women and persons with OA [16–18, 25, 27, 28]. Regarding population, eligible policies were aimed at clinicians (e.g. family physicians, nurse practitioners, rheumatologists, physical/occupational therapists, registered massage therapists, community pharmacists) or decision-makers (e.g. healthcare executives, managers, leaders), and pertained to prevention, diagnosis, treatment, management or support of adults aged 18 + with OA. While our focus was equitable OA care for diverse women, preliminary exploratory searching revealed few policies specific to women, so we included policies relevant to anyone with OA. Regarding issue, we included English- and French-language Canadian policies that focused on or included OA produced by government (e.g. national, provincial, territorial ministries of health), governmental agencies (e.g. quality councils) or other organizations (e.g. academic consortia). Regarding comparisons, policies included documents labelled as policy, strategy, platform, decision, plan, report, framework or other synonymous term that described, compared or analyzed guidance or recommendations for the planning, funding, organization, delivery or improvement of healthcare programs or services. Regarding outcomes, we adopted a broad scope, including policies that identified problems and/or solutions. We excluded clinical guidelines and health technology assessments, which largely focus on recommendations for front-line care, and policies that focused only on rheumatoid, inflammatory or juvenile arthritis. We also excluded documents that solely offered an inventory of existing programs or resources.

### Searching and screening

Additional File S2 describes the comprehensive search strategy in detail. In brief, we searched Canadian government web sites and Google to identify potentially relevant Canadian policies from May 18 to June 29, 2022. AA and MT independently conducted searches, and together compiled results in an Excel file, noting organization, document title and web site address, then independently screened full-text documents against eligibility criteria, and consulted with ARG to resolve discrepancies through discussion. To compile policies, we visited each federal, provincial and territorial government web site to both browse the navigation system (e.g. reports or publications menu item) and search the web site using the keywords “osteoarthritis or arthritis”. By browsing the navigation system and searching for the broader term “arthritis”, we identified any relevant policies labelled with other terms referring to joint disease. In Google, we executed multiple searches using the keywords “arthritis or osteoarthritis” alternately combined with policy, decision, plan, report, guide, framework or strategy, and scanned 60 results for each search combination, after which relevancy of the search results diminished. We also screened the references in all eligible policies.

### Data collection

With respect to deductive content analysis, we extracted data on policy characteristics, strategies to support person-centred OA care and strategies to support equitable access to OA care. Characteristics included year of publication, publishing organization, document structure (e.g. number of pages, sections), policy objective, target audience and methods used to develop the policy (Additional File S3). Person-centred care (PCC) referred to content related to the components of an existing PCC framework that we chose because it was rigorously developed [25], more elaborate than other general PCC frameworks [26], inclusive of approaches deemed essential to person-centred OA care [16–18] and found to be relevant through our prior work on what constitutes PCC for diverse women [27, 28]. The framework we employed includes six domains: foster a healing relationship, exchange information, respond to emotions, manage uncertainty, share decisions and enable self-management (Additional File S4). Equitable access referred to any mention of OA prevalence by intersectional factors (e.g. gender, age, ethno-cultural group, socio-economic status or other vulnerable group), barriers of OA care by intersectional factors, or strategies at any level (e.g. patient, clinician, organization, system) needed or recommended to improve access to person-centred OA care for any equity-seeking group including but not limited to persons by age, sex/gender, geographic location, socioeconomic status or ethno-cultural group, which refers to ethnicity or country of origin (Additional File S5). As a pilot test, AA, MT and ARG independently extracted data from three policies, then compared and discussed results to establish a shared understanding of data extraction. Thereafter, AA extracted data from remaining policies, periodically consulting with MT and ARG to resolve uncertainties. We did not assess the quality of policies.

### Data analysis

With respect to summative content analysis, we used summary statistics to describe policy characteristics, and the number of policies that included mention of the prevalence of OA or barriers to OA care by intersectional factors, person-centred OA care, strategies to improve equitable access to OA care, and research recommendations. We summarized data in tables and text with exemplar content extracted from policies.

## Results

### Policy characteristics

Additional File S3 includes data on policy characteristics and Table 1 provides a summary.

**Table 1:** Summary of characteristics of included policies

Policy	Year published	Organization type	Pages (n)	Methods	Objective
Arthritis Society [29]	2021	Charity	16	Key informant consultation	Recommendations
Alberta Health Services [30]	2020	Government	43	Key informant consultation	Strategic plan
Bone and Joint Canada [31]	2019	Knowledge translation	19	Key informant consultation	Recommendations
Alberta Bone and Joint Health Institute [32]	2019	Government	27	Key informant consultation	Summary of data
Health Quality Ontario [33–35]	2018	Government	58 17	Key informant consultation	Recommendations
Bone and Joint Canada [36, 37]	2014 2015	Knowledge translation	29 13	Key informant consultation	Recommendations
Government of Newfoundland and Labrador Department of Health and Community Services [38]	2012	Government	16	Key informant consultation	Strategic plan
Health Council of Canada [39]	2012	Knowledge translation	60	Not reported	Recommendations
Arthritis Alliance of Canada [40]	2012	Consortium	49	Review of past reports	Strategic plan
Arthritis Alliance of Canada [41]	2011	Consortium	51	Key informant consultation	Recommendations
Government of Newfoundland and Labrador Department of Health and Community Services [42]	2011	Government	20	Review of existing reports	Strategic plan
Arthritis Alliance of Canada [43]	2006	Consortium	69	Key informant consultation	Recommendations
Institute for Clinical Evaluative Sciences (ICES) Toronto [44]	2004	Academic	146	Developed using routinely collected health data	Summary of data
Arthritis Consumer Experts, Arthritis Research Centre of Canada, Canadian Arthritis Patient Alliance [45]	No date	Patient advocacy	6	Not reported	Recommendations

We included 14 policies ranging from 6 to 146 pages published from 2004 to 2021 [29–45]. One policy [33] spanned 3 documents [33–35] and another spanned 2 documents [36, 37]. They were developed by government (5, 35.7%), knowledge translation (3, 21.4%), multi-sector (3, 21.4%), charitable (1, 7.1%), academic (1, 7.1%) or patient advocacy (1, 7.1%) organizations. Most policies addressed arthritis in general (10, 71.4%) and 4 (28.6%) were specific to OA. Policy objectives were to outline recommendations (8, 57.1%), strategic plans (4, 28.5%) or summarize data (2, 14.2%). Policy topics included OA awareness, prevention, diagnosis and/or management (9, 62.3%), wait times for joint replacement surgery (4, 28.6%) or OA self-management (1, 7.1%). Twelve (85.7%) policies were based on key informant consultation (9, 64.2%), review of prior research or reports (2, 14.2%) or routinely-collected health data (1, 7.1%). No policies were specific solely to women.

### Person-centred OA care

Additional File S4 includes all data extracted on person-centred care and Table 2 provides a summary. Among 14 policies, 5 (35.7%) made no reference to PCC [32, 38, 41, 44, 45] and 9 (64.2%) included content related to at least one PCC domain [29–31, 33–37, 39, 40, 42, 43]. In those 9 policies, the most frequently included domains were: enable self-management (7, 50.0%), share decisions (6, 42.8%), and exchange information (4, 28.5%). Few policies addressed the domain of respond to emotions (2, 14.3%). No policies included content for the domains foster a healing relationship and manage uncertainty.

**Table 2:** Summary of content on person-centred OA care in included policies

PCC domain [25, 27, 28]	Policies (n, %) [references]	Examples	
		Limited	Expanded
**Foster a healing relationship** Extend friendly greeting, make eye contact, speak in respectful manner, avoid judgmental attitude.	(0, 0)	--	--
**Exchange information** Listen to concerns, prompt for additional details, understand needs, goals, circumstances and preferences, use lay language, ensure privacy	(4, 28.5) [29, 33, 40, 43]	Despite the prevalence of arthritis…OA is [often] discussed in dismissive terms (e.g., “it’s just age,” or “it’s just wear and tear”). Even the use of the term “elective” can be rather dismissive and should instead always be called “scheduled.” This impacts the timely utilization of publicly available disease prevention resources (e.g., obesity, physical inactivity, knee injury prevention), early diagnosis and treatment interventions in primary care/interdisciplinary care, and investment in research to slow progression of OA [29 p5]	Health care professionals should talk to patients about how osteoarthritis affects energy, mood, sleep, work, hobbies, family, and social life” [33 p14]
**Respond to emotions** Actively inquire about feelings, acknowledge concerns, express empathy, note that such feelings are normal or common, suggest strategies to cope or mitigate emotions	(2, 14.3) [30, 39]	Tackle the social and emotional issues that often accompany chronic disease through referral to a counsellor [39 p24]	Addressing individual mental and psychological characteristics are important [for self-management]: What are the best ways to self-motivate? How do acute injuries and long-term conditions affect mood? How do bone and joint health practitioners collaborate with their mental health colleagues to increasingly provide a whole person, whole health approach [30 p12]
**Manage uncertainty** Offer rationale for tests or treatment, describe likelihood of risks and benefits using words, statistics or pictures	(0, 0)	--	--
**Share decisions** Describe treatment or management options, assess interest in shared decisions, provide information to enable shared decisions, suggest factors to consider in making decisions	(6, 42.8) [30, 33, 39–42]	Patients will be engaged in collaborative and shared decision making, and will be partners in their care [30 p19]	Underpinning self-management support is shared decision-making between patients and health care providers. Health care providers are to provide patients with complex information in clear and understandable terms and, when needed, to help patients develop skills in making decisions that support their physical and mental health. For example, patients should know and be empowered to ask, whenever a new medication is introduced, “How will this new drug interact with my other medications? [39 p21]
**Enable self-management** Set expectations for follow-up care, offer advice on self-care, provide take-home information, refer to other sources of information or support	(7, 50.0) [31, 33, 36, 39, 40, 42, 43]	Enable self-management by providing on-line and hard copy educational materials for individuals living with arthritis and health care providers [40 p24]	Work with people with osteoarthritis to support the development of an individualized, goal-oriented self-management plan that gives the person information and advice on the ongoing management of their symptoms and directs them to resources and other supports they may need. [Plans] should include information about how to access local services, such as exercise classes, weight-management programs, and support groups. [Plans] will also need to consider any other medical conditions you have that may impact your goals and abilities. Depending on [the patients] needs, [plans] might also include information about aids and devices such as suitable shoes, leg braces, orthotics, and hand grips. These things can help you stay active and function well [33 p20]

Even when mentioned, much of this content was brief or vague, offering little to no concrete description of what should be done to achieve PCC for equity-seeking persons with OA including diverse women or other vulnerable groups. For instance, for the most frequently addressed domain of enable self-management, one policy stated: “Enable self-management by providing on-line and hard copy educational materials for individuals living with arthritis and health care providers” [40]. In contrast, a more informative policy stated: “Work with people with osteoarthritis to support the development of an individualized, goal-oriented self-management plan that gives the person information and advice on the ongoing management of their symptoms and directs them to resources and other supports they may need. [Plans] should include information about how to access local services, such as exercise classes, weight-management programs, and support groups. [Plans] will also need to consider any other medical conditions you have that may impact your goals and abilities. Depending on [the patients] needs, [plans] might also include information about aids and devices such as suitable shoes, leg braces, orthotics, and hand grips” [33].

### Equitable access to OA care

Additional File S5 includes all data on OA prevalence and barriers of OA care by intersectional factors, and Table 3 provides a summary. Among 14 policies, 7 (50.0%) acknowledged greater prevalence of OA among particular equity-seeking groups: older age (4, 28.5%), gender, referring to women (5, 35.7%), Indigenous (4, 28.5%) and lower socioeconomic status (2, 14.2%). No policies noted that the burden of OA is greater among racialized or immigrant women [6–9]. Even when policies mentioned the disproportionate burden of OA on diverse persons, details were limited; for example: “while both men and women get arthritis, two thirds of those affected in Canada are women” [40] refers to the higher prevalence of OA among women but does not elaborate on which types of women, acknowledge disparities in access to care, or how to tailor care for diverse women.

**Table 3:** Summary of content acknowledging prevalence of OA and barriers to OA care by intersectional factors

**Factors**	**Policies (n, %)** [references	Examples	
		Limited	Expanded
**Prevalence**			
Older age	(4, 28.5) [33–36, 41, 44]	[Osteoarthritis] is more common in middle to older age (prevalence is 35% in those aged 80 years and older)…[33–35 p3]	As the Canadian population ages, OA prevalence is expected to increase and be highest among those over the age of 70 years. In 2010, approximately 49% of seniors over the age of 70 years are expected to be living with symptomatic OA. By 2040, this number is expected to increase to 71% [41 p31]
Gender	(5, 35.7) [33–36, 40, 41, 44]	While both men and women get arthritis, two thirds of those affected in Canada are women [40 p9]	Arthritis was reported more frequently by women, older people, and people with lower levels of education and lower incomes. These findings are consistent with other surveys, suggesting that people who have arthritis may have fewer resources to deal with the consequences of this condition. The higher prevalence of arthritis among women also raises questions of whether targeted initiatives are necessary to meet the needs of this sector of the population [44 p35]
Indigenous	(4, 28.5) [40, 42–44]	Chronic disease and risk factor rates tend to be even higher among Indigenous people [42 p4]	Arthritis is up to two-and-a-half times as common in the Indigenous community living off reserve (Public Health Agency of Canada, 2003) as in non-Indigenous Canadians. Overall, 27% of Indigenous people living off reserve have arthritis compared with 16% of the general Canadian population. However, arthritis receives little attention as a significant health issue within the Indigenous community [43 pI]
Socio economic status	(2, 14.2) [42, 44]	People with higher levels of education were less likely to report arthritis [44 p22]	Chronic disease becomes more common as people get older. Low incomes, poor social supports, and unhealthy physical environments, also influence the development of chronic disease. These factors combined can contribute to a decreased quality of life for individuals [42 p2]
**Barriers**			
Age	(5, 35.7) [33–35, 49, 40, 43, 44]	Middle-aged and older adults with OA report that their condition has a particularly devastating impact on employment, community mobility, heavy housework, leisure activities, social activities and close relationships [40 p10]	Canadians who have chronic conditions and who are in fair-to-poor health are more likely than the general public to be poorer, older, less educated, and living in rural areas. Advice to join a gym or eat healthier food may be very challenging for these people to comply with [39 p9]
Gender	(3, 21.4) [33–35, 42, 44]	Patient factors such as age, sex, obesity, comorbidities, etc. should not be barriers to referral for joint surgery [33–35 p46]	Women and people with less education and/or lower income were more likely to have potential unmet need for total joint replacement [44 p107]
Ethno-cultural group	(3, 21.4) [33–35, 34, 39, 40]	Numerous studies have identified difficulties in recruiting certain groups for chronic disease self-management programs (ethnic minorities, indigenous communities, rural residents, older people, and people with low income or lower education) and have raised concern that participation tends to drop off as the course progresses [39 p15]	Specific population groups, such as Indigenous peoples, newcomers, refugees, and the homeless, face barriers in access to care. Factors affecting this lack of access include a lack of programs and self-management resources in different languages, as well as culturally safe care. Some of these population groups are also disproportionately affected by poverty, social isolation, and precarious employment; this, in turn, may impact access to effective osteoarthritis care [34 p3]
Socio economic status	(7, 50.0) [32–35, 39, 40, 42–44]	Social, economic, and environmental conditions influence a person’s ability to maintain good health, prevent chronic disease and manage the complications of disease. All determinants of health must be considered to achieve optimal health [42 p6]	One of the challenges for patients navigating conservative treatment is that most of the treatment modalities fall outside of the publicly funded health system. Community educational programs, lifestyle coaching, exercise programs, physiotherapy, massage therapy, and dietary consultations are mostly privately funded. This results in inequity for patients unable or unwilling to fund these treatments out of pocket or who lack adequate private insurance coverage. This situation also leads patients who are financially able to entertain treatment options that are not based on scientific and medical evidence…the result is a complex navigation challenge where patients are forced to become subject matter experts in managing their disease. They are often faced with the difficult choice of paying out of pocket for privately-funded treatments of uncertain benefit, suffering with untreated joint pain until the disease progresses to end-stage, or aggressively pursuing scarce public services, with little guidance or information to assist in their decision-making [32 p6]
Geography	(5, 35.7) [34, 39, 40, 42, 44]	Barriers to program participation [can include] low literacy, disabilities, transportation costs, distances to services, and access to plain language health [42 p9]	For many, gaining access to the right care and the right provider is a challenge. This is particularly true for people living in rural and remote areas, especially [Indigenous] populations, where distance and transportation costs are additional barriers [40 p22]

Nine (64.2%) policies mentioned that intersectional factors contribute to barriers in accessing OA care: lower socioeconomic status (7, 50%), geography, referring to persons living in rural or remote areas (6, 42.8%); older age (5, 35.7%); gender, referring to women (3, 21.4%); and ethno-cultural group (3, 21.4%). Policies provided limited detail about inequitable access to OA care for these diverse groups; for example: “Patient factors such as age, sex, obesity, comorbidities, etc. should not be barriers to referral for joint surgery” [33–35].

### Strategies to improve access

Additional File S5 includes data on strategies recommended in included policies to improve access to OA care, and Table 4 provides a summary. Only 4 (28.6%) policies recommended one or more strategies needed to improve access to OA care. Three of those policies mentioned patient-level strategies: translate self-management educational material into various languages [33–35, 42] and ensure content is culturally-relevant [33–35, 39]. One policy included a clinician-level strategy: incorporate arthritis-related curriculum in the curriculum for training healthcare professionals [40]. Two policies identified 3 system-level strategies: formally assess the equity of OA programs or services in healthcare organizations [33–35], engage lay health leaders representing equity-seeking communities to deliver self-management programs [33–35, 39] and enhance the accessibility of self-management programs via telephone and online support [39].

**Table 4:** Strategies recommended to improve access to OA care

Strategy level	Strategy type	Policies (n,%) [references]	Examples
**Patient** Offered to persons with OA to improve knowledge, confidence, behaviour, OA symptoms, OA status, or quality of life	Translated versions of educational materials	2 (14.2) [33–35, 42]	Healthcare organizations/professionals] should work with community organizations to leverage expertise in languages spoken most frequently in a specific region, and translate existing educational resources on osteoarthritis into these languages [33–35 p12]
	Self-management programs	2 (14.2) [33–35, 39]	Target underserved populations (e.g., ethnic minorities, indigenous communities, rural residents, older people, and people with low income or lower education that tend to have lower participation in programs) through: (1) working in partnership with community health workers, elders, or existing multicultural services to **develop culturally appropriate program materials in terms of language and traditional beliefs about health**, (2) removing practical barriers to participation by offering phone-based interventions, (3) training lay health workers from underserved communities to deliver self-management support, and (4) delivering programs online, but also considering that people with financial or literacy challenges may not have access to a web-enabled device or be comfortable using it [39 p15]
**Clinician** Offered to healthcare professionals to improve knowledge, confidence, behaviour, or how they provide OA care (e.g., skills)	Education (curriculum of healthcare professionals)	1 (7.1) [40]	Government, professional colleges and regulatory agencies, and arthritis stakeholders must focus their efforts and collaborate on the following strategies…Incorporate arthritis-related curriculum into post-graduate and specialty programs that address the needs of vulnerable groups [40 p19]
**System** Developed and/or offered by health systems or government to improve access to OA care, advice and support	Evaluate the equity of programs or services in healthcare organizations	1 (7.1) [33–35]	Health Equity Impact Assessments should be employed [in healthcare organizations] to reduce health disparities between population groups [33–35 p5]
	Train and mobilize lay health leaders	2 (14.3) [33–35, 39]	Target underserved populations (e.g., ethnic minorities, indigenous communities, rural residents, older people, and people with low income or lower education that tend to have lower participation in programs) through…training lay health workers from underserved communities to deliver self-management support [33–35 p46]
	Enhance accessibility of self-management programs via telephone and Internet	1 (7.1) [39]	Target underserved populations (e.g., ethnic minorities, indigenous communities, rural residents, older people, and people with low income or lower education that tend to have lower participation in programs) through: (1) working in partnership with community health workers, elders, or existing multicultural services to develop culturally appropriate program materials in terms of language and traditional beliefs about health, (2) removing practical barriers to participation by **offering phone-based interventions**, (3) training lay health workers from underserved communities to deliver self-management support, and (4) **delivering programs online**, but also considering that people with financial or literacy challenges may not have access to a web-enabled device or be comfortable using it [39 p15]

### Recommended research

Table 5 summarizes research recommendations extracted from 12 (85.7%) included policies. We did not initially intend to extract such data, but content analysis revealed research recommendations, which may be important to address gaps in policies identified by this study. Only 5 (35.7%) policies included research recommendations that explicitly acknowledged equity-seeking groups [30, 32, 40, 41, 45]: Increase investment in all aspects of OA research [40, 43]; Establish priorities for research that improves OA prevention and care [41]; Explore unique OA risks and OA care needs of various equity-seeking groups [30, 32, 40, 41]; Investigate how to support self-management among various equity-seeking groups [30, 32, 45]; Evaluate access to and impact of existing OA management programs to identify factors that contribute to beneficial outcomes [31, 33–37, 42–44]; Identify supports needed by healthcare professionals to foster patient self-management [39]; and Engage persons with OA in research [40].

**Table 5:** Research recommendations extracted from included policies

Policy	Research recommendations
Alberta Health Services, 2020 [30]	− Partner with multiple (clinical networks) and Primary Health Care practitioners to better understand the unique needs of specific populations, such as members of indigenous communities and new arrivals to Canada relating to their joint health. Develop and test projects to bridge the gaps (p 26) − Test multiple conservative management strategies to empower and support patient self-management of OA (p 26) − Partner with researchers, clinicians, and patients to better understand the unique intersection and interplay between obesity and OA including the development of risk-management strategies to better identify and manage obesity-related risk in the surgical orthopedic population and a framework to provide the most appropriate MSK care for patients living with obesity and osteoarthritis (p 25)
Bone and Joint Canada, 2019 [31]	Evaluate current projects related to management of patients with hip and knee OA, to identify learnings and success factors (p 11)
Alberta Bone and Joint Health Institute, 2019 [32]	− Reduce demand for arthroplasty by engaging with researchers, clinicians, and patients to better understand the unique intersection and interplay between obesity and OA (pg.18). − Test multiple conservative management strategies to empower and support patient self-management. There is currently weak evidence for the effectiveness and appropriateness of OA conservative treatments and therapies, leaving many patients unsure of how to proceed in preventative care and management of the disease. Many of the programs and therapies appropriate for supporting OA management are under-utilized as patients often seek out publicly funded services to avoid out of-pocket patient expenses (p 18)
Health Quality Ontario, 2018 [33–35]	Extend collection of patient-reported outcomes to other health care settings, including primary care, to facilitate the delivery of more patient-centred, responsive care (33 p 13)
Bone and Joint Canada, 2014–2015 [36, 37]	Conduct research to evaluate any implementation and/or program transfer/expansion opportunities identified and/or develop an evaluation framework to ensure programs are being developed to meet the needs of individuals with OA across Canada (36 p 15)
Health Council of Canada, 2012 [39]	Continued research and evaluation of strategies for clinicians to foster self-management and engage patients should be supported (p 45)
Arthritis Alliance of Canada, 2012 [40]	− Invest more in OA research to enhance understanding on causes progression, prevention and treatment of arthritis and to develop and implement more effective prevention and care strategies for the future (p 15) − Promote greater networking, collaboration and stakeholder engagement in research, including meaningful participation by individuals living with arthritis (p 18) − Increase investment in arthritis research, [including] (1) Develop a long-term plan to increase research investment across all four pillars of research to levels adequate to better address the economic and social burden of the disease; and (2) Concerted effort to assist current funders in increasing funds raised for arthritis research, as well as engage new funders (p 17) − Enhance knowledge translation and exchange efforts about arthritis prevention, self-management and the effectiveness and efficiency of arthritis care (p 18)
Arthritis Alliance of Canada, 2011 [41]	− While being obese has long been recognized as a risk factor for OA, especially knee OA, the importance of strategies to reduce obesity cannot be underestimated. Research is urgently needed in this area (p 42) − Develop a national framework by establishing research priorities and strategies to support ongoing improvements in the quality of arthritis care and prevention (p 44)
Government of Newfoundland and Labrador Department of Health and Community Services, 2011 [42]	Information about the prevention and management of chronic disease can be collected through research, audits of individual charts, reviews of programs and services, and interviews with individuals, families and caregivers. Can assist policy makers and health care providers to take appropriate actions and develop relevant programs and services to provide better care for individuals (p 15)
Arthritis Alliance of Canada, 2006 [43]	− (1) Determine current access to available effective therapies for arthritis, (2) develop a proposal for the development of a national drug program to ensure rapid and equal access to life-saving and quality-of-life saving medications, and (3) pilot-test a limited expanded access program (p 23) − Governments must invest urgently in research to evaluate risk factors for sport and recreation injury, with subsequent development and testing of interventions designed to ameliorate identified risk factors (p 26)
Institute for Clinical Evaluative Sciences (ICES) Toronto, 2004 [44]	− Future directions for research include: (1) An improved understanding of access to and the quality of primary care for MSK conditions; (2) Ways to improve the organization and coordination of multidisciplinary arthritis care; and, and (3) The relationships between provision of specialty arthritis services and their use, and ways to improve primary care and access to specialty care for MSK conditions (p 82) − Develop, implement and evaluate a chronic disease model of care that includes disease prevention, health promotion, self-management, and is grounded in best practices. The model should incorporate a collaborative network of health professionals, the key principles of client-centredness, and timely and relevant interventions in a variety of settings (p xvii)
Arthritis Consumer Experts, Arthritis Research Centre of Canada, Canadian Arthritis Patient Alliance, No date [45]	In collaboration with “aging in place” experts and the arthritis community alliance, develop an “aging in place” [a concept that sees seniors continuing to live their primary residence with visiting homecare and living space modifications] strategy for those living with chronic disability due to arthritis (p 3)

## Discussion

We analyzed the content of 14 national or provincial Canadian policies issued between 2004 and 2021 by government, knowledge translation, charitable, academic, patient advocacy and multi-sector organizations relevant to OA (*n* = 4) or to arthritis in general including OA (*n* = 10). No policies comprehensively addressed all PCC domains, and few or no policies addressed any of the six domains. Even when mentioned, content was brief, offering little guidance for what should be done to achieve person-centred OA care. Few policies acknowledged greater prevalence of OA among women, older persons, Indigenous persons and those of lower socioeconomic status; or barriers to OA care experienced by those of lower socioeconomic status, in rural or remote areas, of older age, of ethno-cultural groups or women. Only 4 (28.6%) policies recommended strategies needed to improve access to OA care.

Other research also identified a lack of OA-relevant policies in other countries, and limited guidance in those policies for health system reform need to improve access to and quality of OA care. An analysis of government policy and review of published research revealed that a national policy for OA care was established along with evidence-based clinical guidelines following designation of OA as a national health priority in Australia in 2002 but identified only two examples of how the policy or related guidelines influenced service delivery [46]. The authors concluded that, despite clinical guidelines, healthcare professionals are poorly supported by service models to optimize OA care. More recently, a 2023study that compared national policies on musculoskeletal health, including 41 policies that most commonly addressed pain, occupational health, inflammatory conditions and OA, and derived a framework of 47 principles to guide OA policy organized in 8 domains: service delivery, workforce, medicines and technologies, financing; data and information systems, leadership and governance; citizens, consumers and communities; and research and innovation [47]. However, neither of these studies specifically focused on equitable access to person-centred OA care, so our study is unique in this regard. Apart from these two studies of OA-relevant policy, system-level OA research is sparse, as other research has largely focused on the effectiveness of interventions used to promote the uptake of OA clinical guideline recommendations by clinicians [48, 49]. In this study, few policies acknowledged greater prevalence and severity of OA among women, or socio-gendered barriers of access to OA care among diverse women with OA. This finding is similar to our prior analysis of policies relevant to depression and cardiac rehabilitation, conditions with known gendered inequities, which found that few policies acknowledged barriers to care experienced by women or included strategies to reduce those disparities [50].

Several implications emerge from these findings. In Canada, there is a need for national and provincial/territorial policies that recognize the importance of person-centred OA care and include concrete system-level strategies to ensure that all persons with OA access the care they need, particularly equity-seeking groups including women. Given a lack of such policies or related health system reforms in many high-income countries [46, 47], this need may be widespread. Policies could perhaps be improved by including strategies to enhance access to OA care that may be available in the considerable evidence on early diagnosis and management of OA collated in clinical guidelines [3, 4]. Furthermore, information to guide the consideration of PCC in OA policies can be drawn from existing general PCC frameworks [25, 26] and quality indicators of person-centred OA care generated by various groups [16–18]. Hence, efforts may be needed to examine why policies supporting access to early, person-centred OA care are not available. This study revealed some insight on strategies that could be promoted through policy to transform the way that OA care is organized and delivered so that it is accessible to all at the patient (e.g. self-management education material in different languages and tailored to cultural norms), clinician (e.g. healthcare professional education in OA) and system level (e.g. evaluate the equity of OA programs/services, engage lay health leaders in delivering self-management programs, and offer self-management programs in a variety of formats such as telephone and online). However, additional primary research is likely needed to identify additional multi-level strategies; for example, via interviews with diverse persons with OA, clinicians, and health system leaders and policy-makers.

Once such research is generated, it may be difficult to integrate it into policy. Numerous factors challenge the translation of research into policy including perceptions about research evidence, competing influences and practical constraints, requiring dedicated action to promote research findings to policy-makers [51]. A systematic review of 19 studies on how to promote evidence-informed policy-making revealed that tailored policy briefs, workshops, ongoing technical assistance, and sharing of digital instructional materials influenced public health policy [52]. The same review showed that influence on policy was more likely when supported by a series of actions that included establishing an imperative for practice change, building trust between stakeholders and developing a shared vision, describing change mechanisms, using effective communication strategies and providing resources to support policy development.

Although this study investigated whether OA-relevant policies considered any equity-seeking group, our primary interest is in reducing known disparities in OA care among diverse women who are disproportionately impacted by OA [2, 5–11]. This study found that few policies noted a higher prevalence of OA among women or that gender was a barrier to accessing OA care, and no policies included strategies specifically aimed at improving access to person-centred OA care for women. Some insight on approaches to reduce socio-gender inequities in OA care could be gleaned from our prior research involving interviews with women and clinicians [53–55], which formed the basis of national consensus on priorities to improve women’s healthcare experiences and health [56]. As recommended in a few included policies, ongoing research in the OA context must be undertaken to identify strategies that address the needs and preferences of diverse women as well as other equity-seeking groups. For example, a qualitative study was conducted to understand the lived experience of Aboriginal and Torres Strait Islander people with OA, knowledge essential to developing policies that guide culturally-safe care [57]. Another way to ensure that policies reflect the perspectives of equity-seeking groups is to engage them in policy development, as was done in Australia when developed a system-wide model of care for hip and knee osteoarthritis [58]; and in another study that generated consensus among women with OA and healthcare professionals on multi-level strategies need to improve equitable, person-centred OA care for diverse women [59].

This study featured several strengths. We used rigorous methods for content analysis [23, 24], multiple individuals independently conducted screening and data extraction to optimize reliability, and we employed an established PCC framework that encompasses OA-specific elements of PCC to organize and interpret results [16–18, 25]. The entire research team, including a 13-member advisory group of diverse women with OA, were engaged throughout to guide data collection and analysis, and reviewed the findings. Although we are interested in women’s health, we extracted data on any equity-seeking group mentioned in included policies to broaden study relevance. A few limitations should be mentioned. We included documents that others might not consider policy, but we purposefully defined policy broadly to cast a wide net and include as many documents as possible. The search strategy we employed may not have identified all relevant policies. All included policies were relevant to the Canadian context so findings may not be transferrable to other countries with different population health profiles or healthcare systems.

## Conclusions

Canadian OA-relevant policies lack guidance to overcome disparities in access to person-centred OA care for equity-seeking groups including women. This study identified several ways to strengthen policies: explicitly acknowledge disparities in access to and quality of OA care for equity-seeking groups; include detailed guidance for person-centred OA care across all domains (foster a healing relationship, exchange information, respond to emotions, manage uncertainty, share decisions and enable self-management); and offer concrete multi-level (patient, healthcare professional, system) strategies to enhance or transform the way that OA care is organized and delivered to ensure that all persons with OA access the care they need. To do this, ongoing research must identify the needs and preferences of equity-seeking persons with OA, and evaluate the impact of various models of service delivery, knowledge needed to influence OA-relevant policy.

### Electronic supplementary material

Below is the link to the electronic supplementary material.

Supplementary Material 1

Supplementary Material 2

Supplementary Material 3

Supplementary Material 4

Supplementary Material 5

## Data Availability

All data generated or analysed during this study are included in this published article and its supplementary information files.

## Abbreviations

OA: Osteoarthritis
PCC: Person-centred care

## References

[1] Kraus VB, Blanco FJ, Englund M, Karsdal MA, Lohmander LS (2015). Call for standardized definitions of osteoarthritis and risk stratification for clinical trials and clinical use. Osteo Cartil.

[2] Long H, Liu Q, Yin H, Wang K, Diao N, Zhang Y (2022). Prevalence trends of site-specific osteoarthritis from 1990 to 2019: findings from the global burden of disease study 2019. Arthritis Rheum.

[3] Badley EM, Wilfong JM, Zahid S, Perruccio AV. The status of arthritis in Canada. In: National report. Arthritis Society. 2019. https://arthritis.ca/getmedia/13aff08f-f206-4c6e-a709-beb80b97bd51/ACREU_Arthritis-Society_National-Report-2019_final.pdf. Accessed 26 July 2022.

[4] Kolaskinski SL, Neogi T, Hochberg MC, Oatis C, Guyatt G, Block J (2021). 2019 American College of Rheumatology/Arthritis Foundation guideline for the management of osteoarthritis of the hand, hip, and knee. Arthritis Care Res (Hoboken).

[5] Marshall DA, Liu X, Barnabe C, Yee K, Faris PD, Barber C (2019). Existing comorbidities in people with osteoarthritis: a retrospective analysis of a population-based cohort in Alberta, Canada. BMJ Open.

[6] Hawker GA, Badley EM, Jaglal S, Dunn S, Croxford R, Ko B et al. Musculoskeletal conditions. In: Project for an Ontario Women’s Health Evidence-Based Report (POWER) Study. POWER Report. 2010. https://powerstudy.ca/power-report/volume2/musculoskeletal-conditions/. Accessed 26 July 2019.

[7] Cavanaugh AM, Rauh MJ, Thompson CA, Alcaraz J, Mihalko WM, Bird CE (2019). Racial and ethnic disparities in utilization of total knee arthroplasty among older women. Osteoarthr Cartil.

[8] Schwartzberg HG, Roy R, Wilson K, Starring H, Leonardi C, Bronstone A (2021). Patient characteristics independently associated with knee osteoarthritis symptom severity at initial orthopedic consultation. J Clin Rheumatol.

[9] Odonkor CA, Esparza R, Flores LE, Verduzco-Gutierrez M, Escalon MX, Solinsky R (2021). Disparities in Health Care for Black patients in Physical Medicine and Rehabilitation in the United States: a narrative review. PM R.

[10] Rabah NM, Knusel KD, Khan HA, Marcus RE (2020). Are there nationwide socioeconomic and demographic disparities in the use of outpatient orthopaedic services?. Clin Orthop Relat Res.

[11] Kenning C, Daker-White G, Blakemore A, Panagioti M, Waheed W (2017). Barriers and facilitators in accessing care by ethnic minority groups: a meta-synthesis of qualitative studies. BMC Psychiatry.

[12] Pan-Canadian Health Inequalities Data Tool. A joint initiative of the Public Health Agency of Canada, the Pan-Canadian Public Health Network, Statistics Canada and the Canadian Institute of Health Information. 2017. https://health-infobase.canada.ca/health-inequalities/data-tool/index. Accessed 26 July 2022.

[13] Abenoja A, Theodorlis M, Ahluwalia V, Battistella M, Borkhoff CM, Hazlewood GS, Lofters A et al. Strategies to improve equitable access to early osteoarthritis diagnosis and management: an updated review. Arthritis Care Res. 2023;online ahead of print: 10.1002/acr.25179. 10.1002/acr.25179 PMC11771570 37382031

[14] Egerton T, Diamond LE, Buchbinder R, Bennell KL, Slade SC (2017). A systematic review and evidence synthesis of qualitative studies to identify primary care clinicians’ barriers and enablers to the management of osteoarthritis. Osteoarthr Cartil.

[15] Mackay C, Hawker GA, Jaglal SB (2020). Qualitative study exploring the factors influencing physical therapy management of early knee osteoarthritis in Canada. Phys Ther.

[16] Hinman RS, Allen KD, Bennell KL, Berenbaum F, Betteridge N, Briggs AM (2002). Development of a core capability framework for qualified health professionals to optimise care for people with osteoarthritis: an OARSI initiative. Osteoarthr Cartil.

[17] Stoffer MA, Smolen JS, Woolf A, Ambrozic A, Berghea F, Boonen A (2015). Development of patient-centred standards of care for osteoarthritis in Europe: the eumusc.net-project. Ann Rheum Dis.

[18] Blackburn S, Higginbottom A, Taylor R, Bird J, Østerås N, Hagen KB (2016). Patient-reported quality indicators for osteoarthritis: a patient and public generated self-report measure for primary care. Res Involve Engage.

[19] Hunter DJ, Neogi T, Hochberg MC (2011). Quality of osteoarthritis management and the need for reform in the US. Arthritis Care Res.

[20] Thomson K, Hillier-Brown F, Todd A, McNamara C, Huijits T, Bambra C (2018). The effects of public health policies on health inequalities in high-income countries: an umbrellareview. BMC Public Health.

[21] Pollack Porter KM, Rutkow L, McGinty EE (2018). The importance of policy change for addressing public health problems. Public Health Rep.

[22] Morency JD, Caron Malenfant E, MacIsaac S. Immigrant and diversity. In:Population projections for Canada and its regions, 2011 to 2036. Statistics Canada. 2017. https://www150.statcan.gc.ca/n1/pub/91-551-x/91-551-x2017001-eng.htm. Accessed 26 July 2022.

[23] Elo S, Kyngas H (2008). The qualitative content analysis process. J Adv Nurs.

[24] Hsieh HF, Shannon SE (2005). Three approaches to qualitative content analysis. Qual Health Res.

[25] McCormack LA, Treiman L, Rupert D, Williams-Piehota P, Nadler E, Arora NK (2011). Measuring patient-centered communication in cancer care: a literature review and the development of a systematic approach. Soc Sci Med.

[26] Scholl I, Zill JM, Härter M, Dirmaier J (2014). An integrative model of patient-centeredness—a systematic review and concept analysis. PLoS ONE.

[27] Nyhof BB, Jameel B, Dunn S, Grace SL, Khanlou N, Stewart DE (2020). Identifying strategies to implement patient-centred care for women: qualitative interviews with women. Patient Educ Couns.

[28] Gagliardi AR, Kim C, Jameel B (2020). Physician behaviours that optimize patient-centred care: focus groups with migrant women. Health Expect.

[29] Arthritis Society. The wait: addressing Canada’s critical backlog of hip and knee replacement surgeries. Arthritis Society. 2021. https://arthritis.ca/getmedia/63f2646b-0c7a-4064-98cf-c1ac8460c326/ArthritisSociety_WaitTimesReport_EN.pdf. Accessed 26 July 2022.

[30] Alberta Health Services. From illness to wellness transformational roadmap 2020–2025. AHS: Bone and Joint Health Strategic Clinical Network. 2020. https://www.albertahealthservices.ca/assets/about/scn/ahs-scn-bjh-roadmap-2020-2025.pdf. Accessed 26 July 2022.

[31] Bone and Joint Canada. Managing hip and knee osteoarthritis in Canada. BJC. 2019. http://boneandjointcanada.com/wp-content/uploads/2019/06/Managing-hip-and-knee-osteoarthritis-in-Canada_Final_June2019.pdf. Accessed 26 July 2022.

[32] Alberta Bone and Joint Health Institute. The osteoarthritis crisis in Alberta: access, quality, and long-term planning. ABJHI. 2019. https://www.albertaboneandjoint.com/wp-content/uploads/2019/10/ABJHI_Osteoarthritis_Crisis_in_Alberta_2019.pdf. Accessed 26 July 2022.

[33] Health Quality Ontario. Osteoarthritis. In: Care for adults with osteoarthritis of the knee, hip or hand. Ontario: HQO. 2018. https://www.hqontario.ca/evidence-to-improve-care/quality-standards/view-all-quality-standards/osteoarthritis. Accessed 26 July 2022.

[34] Health Quality Ontario. Recommendations for adoption: osteoarthritis of the knee, hip, or hand. In: Recommendations to enable widespread adoption of this quality standard. HQO. 2018. https://www.hqontario.ca/Portals/0/documents/evidence/quality-standards/qs-osteoarthritis-recommendations-for-adoption-en.pdf. Accessed 26 July 2022.

[35] Health Quality Ontario. Quality standards. In: Process and Methods guide. HQO. 2016. http://www.hqontario.ca/portals/0/documents/evidence/quality-standards/qs-process-guide-en.pdf. Accessed 26 July 2022.

[36] Davis A, Badley DE, McGlasson R, Alleyne J. Reducing the impact of OA: A report on the prevention and effective management in Canada. In: phase 1: stakeholder engagement phase 2: meeting. BJC. 2014. http://boneandjointcanada.com/wp-content/uploads/2014/05/BJC-OA-Meeting-Report-Final.pdf. Accessed 26 July 2022.

[37] Davis A, McGlasson R. Reducing the impact of OA: prevention and effective management in Canada. In: Phase 3: promotion of effective strategies final report. BJC. 2015. http://boneandjointcanada.com/wp-content/uploads/2021/03/BJC-Phase-3-OA-Report-2015.pdf. Accessed 26 July 2022.

[38] Government of Newfoundland and Labrador. A strategy to reduce hip and knee joint replacement surgery wait times in Newfoundland and Labrador. Department of Health and Community Services. 2012. https://www.gov.nl.ca/hcs/files/wait-times-pdf-orthopedic-wait-times-strategy.pdf. Accessed 26 July 2022.

[39] Health Council of Canada. Self-management support for Canadians with chronic health conditions. In: A focus on primary care. 2012. https://www.selfmanagementbc.ca/uploads/HCC_SelfManagementReport_FA.pdf. Accessed 26 July 2022.

[40] Arthritis Alliance of Canada. Joint action on arthritis a framework to improve arthritis prevention and care in Canada. AAC. 2012. https://www.arthritisalliance.ca/images/PDF/eng/Initiatives/201209171000_framework_EN_588.pdf. Accessed 26 July 2022.

[41] Arthritis Alliance of Canada. The impact of arthritis in Canada: today and over the next 30 years. AAC. 2011. https://www.arthritisalliance.ca/images/PDF/eng/Initiatives/20111022_2200_impact_of_arthritis.pdf. Accessed 26 July 2022.

[42] Government of Newfoundland and Labrador. Improving health together. In: A policy framework for chronic disease prevention and management in Newfoundland and Labrador. Department of Health and Community Services. 2011. https://www.gov.nl.ca/hcs/files/chronicdisease-improving-health-together.pdf. Accessed 26 July 2022.

[43] Alliance for Canadian Arthritis Program. Arthritis isn’t a big deal… until you get it. Ask 4 million Canadians. In: Report from the summit on standards for arthritis prevention and care. 2006. http://www.arthritisalliance.ca/images/PDF/eng/Initiatives/SAPC%20Full%20Report%2020060331%20en.pdf.Accessed 26 July 2022.

[44] Badley EM, Boyle E, Corrigan L, DeBoer D, Glazier RH, Guan J, Hawker G (2004). Arthritis and related conditions in Ontario: ICES research atlas.

[45] Arthritis Consumer Experts, Arthritis Research Centre of Canada, Canadian Arthritis Patient Alliance. Making arthritis care in BC the best in Canada. No date. https://jointhealth.org/pdfs/ArthritisCareInBC.pdf. Accessed 26 July 2022.

[46] Brand C, Hunter D, Hinman R, March L, Osborne R, Bennell K (2011). Improving care for people with osteoarthritis of the hip and knee: how has national policy for osteoarthritis been translated into service models in Australia?. Int J Rheum Dis.

[47] Schneider CH, Parambath S, Young JJ, Jain S, Slater H, Sharma S (2023). From local action to global policy: a comparative policy content analysis of national policies to address musculoskeletal health to inform global policy development. Int J Health Policy Manag.

[48] Al Zoubi FM, Menon A, Mayo NE, Bussieres AE (2018). The effectiveness of interventions designed to increase the uptake of clinical practice guidelines and best practices among musculoskeletal professionals: a systematic review. BMC Health Serv Res.

[49] Zadro JR, O’Keeffe M, Allison JL, Lembke KA, Forbes JL, Maher CG (2020). Effectiveness of implementation strategies to improve adherence of physical therapist treatment choices to clinical practice guidelines for musculoskeletal conditions: systematic review. Phys Ther.

[50] Gagliardi AR, Dunn S, Foster AM, Grace SL, Khanlou N, Stewart DE (2020). Is patient-centred care for women a priority for policy-makers? Content analysis of government policies. Health Res Policy Syst.

[51] Orton L, Lloyd-Williams F, Taylor-Robinson D, O’Flaherty M (2011). Capewell. The use of research evidence in public health decision making processes: systematic review. PLoS ONE.

[52] Sarkies MN, Bowles KA, Skinner EH, Haas R, Lane H, Haines TP (2017). The effectiveness of research implementation strategies for promoting evidence-informed policy and management decisions in healthcare: a systematic review. Implement Sci.

[53] Nyhoff BB, Jameel B, Dunn S, Grace SL, Khanlou N, Stewart DE, Gagliardi AR (2020). Identifying strategies to implement patient-centred care for women: qualitative interviews with women. Patient Educ Couns.

[54] Gagliardi AR, Kim C, Jameel B (2020). Physician behaviours that optimize patient-centred care: focus groups with migrant women. Health Expect.

[55] Filler T, Dunn S, Grace SL, Straus SE, Stewart DE, Gagliardi AR (2020). Multi-level strategies to tailor patient-centred care for women: qualitative interviews with clinicians. BMC Health Serv Res.

[56] Filler T, Foster AM, Grace SL, Stewart DE, Straus SE, Gagliardi AR (2020). Patient-centred care for women: Delphi consensus on evidence-derived recommendations. Value Health.

[57] O’Brien P, Prehn R, Green C, Lin I, Flanagan W, Conley B (2023). Understanding the impact and tackling the burden of osteoarthritis for Aboriginal and Torres Strait Islander people. Arthritis Care Res.

[58] Briggs AM, Page CJ, Shaw BR, Bendrups A, Philip K, Cary B (2018). A model of care for osteoarthritis of the hip and knee: development of a system-wide plan for the health sector in Victoria, Australia. Healthc Policy.

[59] Iziduh S, Abenoja A, Theodorlis M, Ahluwalia V, Battistella M, Borkhoff CM (2024). Priority strategies to reduce socio-gendered inequities in access to person-centred osteoarthritis care: Delphi survey. BMJ Open.

