# Supplementary material for: Policies in Canada fail to address disparities in access to person-centred osteoarthritis care: a content analysis
Source: BMC Health Serv Res. 2024 Apr 25;24:522. doi: 10.1186/s12913-024-10966-5 (PMC11044343; doi:10.1186/s12913-024-10966-5)
Supplement: Supplementary file 1 — Supplementary Material 1 [file 12913_2024_10966_MOESM1_ESM.docx]

**Additional File 1. Eligibility criteria**

**Definition**“Policies” refers to documents, possibly labelled as policy, decision, plan, framework, strategy, or synonymous term, that guide the planning, funding, organization, delivery or improvement of healthcare programs or services. Although policy-related actions might benefit the public, patients, or healthcare professionals, they are often aimed at individuals or organizations that can implement those actions; for example, government at various levels, health system leaders, regional bodies that oversee healthcare delivery or healthcare organization executives or managers, where healthcare organizations include but are not limited to hospitals, professional societies, etc.

**ELIGIBLE**

**People/Population**

- Policies directed to (meaning influence, affect or support) adults aged 18+ with:
  - Arthritis and/or OA, and may be included with other conditions (e.g., chronic diseases)
  - Osteoarthritis affecting specific joints (e.g., policy may be specific to knee or back osteoarthritis)
- The policy can also be directed to clinicians (e.g., family physicians, nurse practitioners, rheumatologists, physical/occupational therapists, registered massage therapists, community pharmacists), or decision-makers (e.g., health care executives, managers, or leaders)

**Issue/Intervention**

- Policies pertaining to the planning, funding, organization, delivery or improvement of healthcare programs or services related to the prevention, diagnosis, treatment, management, or support of persons with osteoarthritis or the healthcare professionals (e.g., physicians of various specialties, nurses, nurse practitioners, physiotherapists, etc.) who care for them
- Can be FOCUSED on or be RELEVANT to arthritis or OA (e.g., the policy may be focused on an aspect of care, service or support related to OA management such as joint replacement, pain management, self-management, or post-operative management)
- Includes Canadian federal, provincial, or territorial policies
- Developed by:
  - Government
  - Governmental ministry of health
  - Agency with government-delegated authority over healthcare delivery or monitoring
  - Other organization or consortium of organizations with a special interest in improving care (e.g., provincial Health Quality Councils, national organizations such as Healthcare Excellence Canada, etc.)
- Published in English or French language (for Quebec)
- No date restrictions

**Comparisons**

- May compare one or more strategies, programs, or services for caring for or supporting persons with osteoarthritis, clinicians who care for them, or the decision-makers who oversee service delivery
- May focus on prevention, diagnosis, treatment, support, or self-management of OA
- Treatments include:
  - First line treatment:
    - Education/counseling about OA and/or about how to self-manage OA (e.g., counselling, courses, information)
    - Encourage increased physical activity
    - Encourage healthy diet
    - Encourage weight loss
    - Pharmacologic and non-pharmacologic means of managing pain
  - Second line treatment:
    - Injections (e.g., corticosteroids, hyaluronic acid)
    - Arthroscopic surgery (minimally invasive procedure to examine and sometimes correct damage in a joint)
    - Osteotomy (surgical procedure to shorten or lengthen a bone to adjust its alignment)
    - Arthrodesis/Joint fusion (fusion of two bones to lessen pain and create more stable joint)
    - Joint replacement (e.g., arthroplasty, of any of the body areas noted above)

**Outcomes**

- Any goals or objectives specified in the policies, but may include:
  - Identification of challenges, problems, or gaps and strategies needed to overcome those barriers
  - Recommendations for accessing, organizing, delivering, or evaluating prevention, diagnosis, treatment, support, or self-management of OA
  - Performance measures (i.e., quality indicators) associated with desired benefits of strategies, programs, or services at the patient, clinician, organization, or health system level
  - Reviews or evaluations of services or programs and corresponding recommendations, descriptions, or priorities for research or programs to address identified gaps

**NOT ELIGIBLE**

- Policies focused **only** on rheumatoid arthritis, inflammatory arthritis, juvenile arthritis
- Documents or reports that are:
  - Clinical guidelines:
    - Documents developed using standardized methods by non-profit organizations (e.g. academic groups, government, governmental agencies, professional societies, charities/foundations) that include recommendations informed by a systematic review of evidence and an assessment of the benefits and harms of alternative care options; used by healthcare professionals to guide their care of patients, or by healthcare managers or policy-makers to inform decisions about organizing and funding healthcare services
    - E.g., <https://www.aaos.org/globalassets/quality-and-practice-resources/glenohumeral/gjo-cpg.pdf>
  - Health technology assessments:
    - Multidisciplinary process used to inform policy and clinical decision making around which devices, diagnostics, medical procedures, and programs should be implemented; addresses the medical, social, economic, and ethical issues related to the use of a health technology
    - E.g., <https://www2.gov.bc.ca/assets/gov/government/ministries-organizations/ministries/health/hip-implants-hta-final.pdf>
    - E.g., <https://www-tandfonline-com.proxy1.lib.uwo.ca/doi/full/10.1057/jos.2014.43>
- Documents or reports that only summarize existing programs or resources but do not offer any recommendations or framework for what is needed to improve OA services, programs, or resources
