# Supplementary material for: Policies in Canada fail to address disparities in access to person-centred osteoarthritis care: a content analysis
Source: BMC Health Serv Res. 2024 Apr 25;24:522. doi: 10.1186/s12913-024-10966-5 (PMC11044343; doi:10.1186/s12913-024-10966-5)
Supplement: Supplementary file 2 — Supplementary Material 2 [file 12913_2024_10966_MOESM2_ESM.docx]

**Additional File 2. Search strategy**

“Policies” refers to documents, possibly labelled as policy, decision, plan, report, guide, framework, strategy or synonymous term, that guide the planning, funding, organization, delivery or improvement of healthcare programs or services, and are produced by government, governmental ministry of health, agency with government-delegated authority over healthcare delivery or monitoring, or other organization or consortium of organizations with a special interest in improving care.

**Government web sites**

Federal

Health Canada: https://www.canada.ca/en/health-canada.html (federal)

Province/Territory

| Province/Territory | Website |
| --- | --- |
| British Columbia | https://www2.gov.bc.ca/ |
| Alberta | https://www.albertahealthservices.ca/ |
| Saskatchewan | [https://www.saskatchewan.ca/](https://www.saskatchewan.ca/%20) |
| Manitoba | https://gov.mb.ca/ |
| Ontario | https://www.ontariohealth.ca/ |
| Quebec | https://www.quebec.ca/ |
| New Brunswick | https://www2.gnb.ca |
| Nova Scotia | https://www.nshealth.ca |
| Prince Edward Island | https://www.princeedwardisland.ca |
| Newfoundland | https://www.gov.nl.ca |
| Yukon | https://yukon.ca/ |
| Northwest Territories | https://www.nthssa.ca/ |
| Nunavut | https://www.gov.nu.ca |

On these websites, perform the following searches:

1. **Search with keywords –** use the search engine to search for: arthritis, osteoarthritis

- Scan the results to assess how many results are potentially relevant on each page (e.g. after the 8^th^ page, results are no longer relevant, stop scanning past page 8)
  - Keep track of how many items are screened per website, as this will be the cut-off we use once we decide on where the relevancy drops off (e.g. the first 8 pages of each website with 10 results on each page = 80)
- Assess title for relevancy; if title unclear, click on each item to assess description/executive summary
  - If the selected item is not a policy, scan through it to see if it links to/references one
  - If the selected item discusses arthritis, scan through it to see if there is a section on osteoarthritis in particular
- For each potentially eligible document:
  - Note the document title, organization/source, and link/URL in an Excel file
  - Download the document as a PDF/Word document and save to a folder on your computer
    - - If unable to download directly, copy and paste the web content into a Word document
      - Name the downloaded files by: organization – year – document title

1. **Browse with navigation –** browse with navigation menu options for policies pertaining to arthritis or osteoarthritis

- Menu options will vary across sites (e.g. may be under “publications” à “chronic diseases” à “arthritis”
- Keep track of the menu options/tabs you peruse in Excel or a Methods document (we will ask you to prepare a detailed description of Methods)
- Keep track of how many items are screened per website, as this will be the cut-off we use once we decide on where the relevancy drops off (e.g. the first 8 pages of each website with 10 results on each page = 80)
  - Note – will likely not have as many items when browsing with navigation as you will when searching with keywords
- Assess title for relevancy; if title unclear, click on each item to assess description
  - If the selected item is not directly a policy, scan through it to see if it links to/references one
  - If the selected item discusses arthritis, scan through it to see if there is a section on osteoarthritis in particular
- For each potentially eligible document:
  - Note the document title, organization/source, and link/URL in an Excel file
  - Download the document as a PDF/Word document and save to a folder on your computer.
    - If unable to download directly, copy and paste the web content into a Word document
    - Name the downloaded files by: province abbreviation, organization, year and brief title (e.g. BC ministry health 2020 arthritis strategy)

**Google searches**

Search Google for:

1. Canada and osteoarthritis
2. Canada and arthritis
   1. For any relevant documents, scan through/CTRL+F for a section on osteoarthritis
3. <province/territory> and osteoarthritis
4. <province/territory> and arthritis
   1. For any relevant documents, scan through/CTRL+F for a section on osteoarthritis
5. Repeat above searches adding keywords: policy, decision, plan, report, guide, framework, strategy

- Scan the results to assess how many results are potentially relevant on each page (e.g. after the 8^th^ page, results are no longer relevant, stop scanning past page 8)
  - Keep track of how many items are screened per Google search, as this will be the cut-off we use once we decide on where the relevancy drops off (e.g. the first 8 pages of each website with 10 results on each page = 80)
- Assess title for relevancy; if title unclear, click on each item to assess description
  - If the selected item is not directly a policy, scan through it to see if it links to/references one
  - If the selected item discusses arthritis, scan through it to see if there is a section on osteoarthritis in particular
- For each potentially eligible document:
  - Note the document title, organization/source, and link/URL in an Excel file
  - Download the document as a PDF/Word document and save to a folder on your computer
    - If unable to download directly, copy and paste the web content into a Word document
      - Name the downloaded files by: province abbreviation, organization, year and brief title (e.g. BC ministry health 2020 arthritis strategy)

Examples:

- Canada AND "osteoarthritis" AND policy OR decision OR plan OR report OR guide OR framework OR strategy
- Canada AND "arthritis" AND policy OR decision OR plan OR report OR guide OR framework OR strategy

*Note – for Quebec, search in English and French; elaborate search strategy to include terms: “arthrose” (French for osteoarthritis) and “arthrite” (French for arthritis). We can translate French documents to English using Deepl: https://www.deepl.com/en/translator
