# Supplementary material for: Policies in Canada fail to address disparities in access to person-centred osteoarthritis care: a content analysis
Source: BMC Health Serv Res. 2024 Apr 25;24:522. doi: 10.1186/s12913-024-10966-5 (PMC11044343; doi:10.1186/s12913-024-10966-5)
Supplement: Supplementary file 3 — Supplementary Material 3 [file 12913_2024_10966_MOESM3_ESM.docx]

**Additional File 3. Characteristics of included policies**

| Policy  [Title, Source, Year, Focus] | Content | Objective | Target audience | Development approach |
| --- | --- | --- | --- | --- |
| THE WAIT: Addressing Canada’s Critical Backlog of Hip and Knee Replacement Surgeries  Arthritis Society  2021 [29]  Arthritis; reducing wait times | 16-page report containing: executive summary, introduction, and 4 sections including 1) an overview of the problem around wait times for hip and knee procedures in Canada, 2) opportunities and solutions, 3) a summary, and 4) next steps. The document also contains an appendix outlining Arthritis Society’s Wait Times Working Group. | Analyzes factors contributing to Canada’s crisis in joint replacement wait times and proposes solutions related to delivering faster, more efficient and more patient-centred hip and knee surgeries to more Canadians (pg.3). | Governments, health system, healthcare providers | A Pan-Canadian Wait Times Working Group was assembled, including clinicians, advocates, and health system leaders across Canada (pg.2,3,15). The Working Group met for 2 sessions to analyze and provide expertise and perspectives on the factors prolonging and exacerbating joint replacement wait times and identify and prioritize a series of solutions indicated in the report to address issues related to hip and knee surgeries (pg.3,4). |
| From Illness to Wellness  Transformational Road Map 2020-2025  Alberta Health Services  2020 [30]  Arthritis; bone and joint health | 43-page report containing: 1) introduction, 2) development of the transformational roadmap outlining the direction and activities of a strategic clinical network, 3) overview of the Bone and Joint Health SCN 2020-2025 Transformational Roadmap, 4) Evidence-Informed Practice, Research & Innovation, 5) Measurement and Evaluation, including next steps, and 6) the Bone and Joint Health team. The document also contains an appendix outlining Bone and Joint Health successes and achievements. | Outlines Bone and Joint Health Strategic Clinical Network strategic plan from 2020-2025, focusing on three interrelated priorities that aim to maximize mobility and functions, mend the loss of function, and mitigate risks for bone and joint injuries and conditions: 1) bone health, 2) joint health, and 3) movement and function (pg.5). | Researchers, administrators, policy makers, change  management experts, patients, and healthcare providers (pg.6). | To develop the Transformational Roadmap, a review of the current state of the musculoskeletal health was undertaken in 2019 with the Core Committee. A series of leadership team and Core Committee meetings were held to help articulate guiding principles and values, and three strategic priority areas. Following this, an extensive consultation process ensued with a wide range of stakeholders directly involved in Alberta’s healthcare system, including patients, physicians, nurses and allied health, administrators, and policy makers. The consultation process included multiple engagement approaches including an online community survey, a series of focus groups and telephone interviews with stakeholder groups, presentations at different conferences and events (pg.16). |
| Managing Hip and Knee  Osteoarthritis in Canada  Bone and Joint Canada    2019 [31]  Osteoarthritis | 19-page report containing: 1) executive summary, 2) project background and rationale, 3) details about the meeting including, goals, attendees, and a summary, 4) recommendations, summary, and next steps, and 5) the meeting agenda. | Address the needs of individuals with hip and knee osteoarthritis and devises recommendations and action items to improve the management of hip and knee osteoarthritis at a local, regional/provincial, and national level (pg. 3,16). | Local, regional/provincial, national level governments across Canada, health care leaders | Report developed through a meeting, framed through a presentation by a patient with OA attended by representatives from the provinces and international and national leaders who gave presentations on status of OA Care – updated research, successful programs, tools for success, and evaluation. Attendees discussed gaps and opportunities to improve care for individuals with hip and knee OA, which are stated as recommendations.    Report also developed from action taken to improve OA management since “Reducing the impact of OA: A report on the prevention and effective management in Canada” phase 1 and 2 and phase 3 and builds on recommendations made in these documents (pg.5). |
| The Osteoarthritis Crisis in Alberta: Access, Quality, and Long-Term Planning  Alberta Bone and Joint Health Institute  2019 [32]  Osteoarthritis | 27-page report containing: 1) executive summary, 2) access to care, including patient experience and managing OA, 3) demand for arthroplasty, 4) ‘taming of the queue’, which includes information related to the access gap and surgery waiting times crisis and has a conclusion. The documents also contain an appendix on methods. | Outlines current wait time crisis, provides important content, estimates specific volumes required to achieve and maintain targets and informs readers of emerging initiatives aimed at addressing some of the factors that contribute to Albertans developing OA (pg.4). | Alberta Health Services Bone and Joint Health Strategic Clinical Network, arthritis stakeholders | Several stakeholders contributed to the development of the report including: Alberta Health Services’ Bone and Joint Health Strategic Clinical Network, 90+ orthopaedic surgeons in Alberta, health services researchers, the Patient and Community engagement Research group, caregivers, Albertans with osteoarthritis (pg.3).  The following models were used to obtain data around demand for arthroplasty: 1) the Alberta Government Interactive Health Data Application using the “Demographics, Population Projections by Zone – 2018-2047” data set, 2) Discharge Abstract Database and operating room information systems, 3) the annual volume of surgical referrals by date of referral, 4) Alberta government demographic projections, 5) clinic EMR data from Calgary, Edmonton, Red Deer, and Lethbridge (pg.26).  Monte Carlo simulation was initially used for waiting time projections, with a simple arithmetic conversion of the projected mean to an estimated 90^th^ percentile using a slowly declining 90^th^ mean ratio ultimately being adopted (pg.27). |
| Osteoarthritis  Care for Adults with Osteoarthritis  of the Knee, Hip, or Hand  Quality Standard  Osteoarthritis  Care for Adults with Osteoarthritis  of the Knee, Hip, or Hand  Recommendations  Health Quality Ontario  2018 [33,34]  Osteoarthritis of the knee, hip, or hand  Also includes:  Quality Standards: Process and Methods Guide  2016 [35] | *Standard*  58-page document containing: information about quality standards, an overview of the 10 quality statements, and 10 sections on each of the quality statements.  *Recommendations*  17-page document containing: recommendations for adoption of the quality standards, a section on measurement and reporting, and 2 appendices on: 1) methods and process for developing recommendations, and 2) a summary of recommendations. | *Standard*  The quality standard focuses on the assessment, diagnosis, and management of osteoarthritis for people across all health care settings and health care professionals to improve care for patients with OA.  *Recommendations*  Summary of recommendations to support the adoption of the quality standard for care at local practice and system-wide levels | *Standard*  Patients, clinicians, health organizations  *Recommendations*  Healthcare providers, organizations, local health integration networks (LHINs), and other health system partners | *Standard*  Developed by Health Quality Ontario in collaboration with an Advisory Committee comprised of clinicians, researchers, and patients; clinical experts; patients; residents; and caregivers across Ontario.  *Process and Methods Guide*  Quality standards development process includes:  -selecting and prioritizing topics via public input, organization input, and government  -determining the scope (i.e., population, care setting) and scoping process, which involves scanning existing clinical practice guidelines and other evidence sources  -engaging partners, health care professionals, patients, families, and caregivers  throughout the development of the quality standard (especially in early stages, and revised with stakeholder input)  -establish Osteoarthritis Quality Standard Advisory  Committee made up of two co-chairs and several individuals from different organizations  -Evidence-based quality statements developed through expertise and feedback from Quality Standard Advisory Committee identified in a series of meetings  -Development of quality indicators  -Consulting the Public to review draft of quality standards  -Implementation plan  -Evaluation and updating quality standards as an ongoing process  *Recommendations*  Recommendations were developed after reviewing evidence on implementation, examining existing programs, and consulting with various stakeholders, including: the Osteoarthritis Quality Standard Advisory Committee, patients with osteoarthritis, healthcare professionals, arthritis organizations, researchers, and the public (pg.15)  Individuals and organizations were further engaged through structured meetings, a public comment period, and targeted interviews to inform the gaps in knowledge, behaviours, and attitudes related to the quality standard (pg.15) |
| Reducing the impact of OA:  A report on the prevention and effective  management in Canada  Phase 1: stakeholder engagement and phase 2: meeting 2014 [36]  Phase 3: Promotion of effective strategies 2015 [37]  Bone and Joint Canada  Osteoarthritis; prevention and management | *Phase 1 and 2*  29-page report containing: 1) acknowledgements, including information on funding and steering committee, 2) background on OA care in Canada, 3) project goal, 4) phase 1: stakeholder engagement, and 5) phase 2  6) challenges and opportunities in an OA model, 7) leaders and partners, 8) action items 9) next steps and 10) appendices on stakeholders and meeting attendees,  meeting agenda, presentation, results of breakout sessions, session feedback, and  references.  *Phase 3*  13-page report containing: 1) acknowledgements, 2) background, including OA care and management and overview of phase 1 and 2, 3) an overview of phase 3 and 4) conclusion, including next steps. The document also contains 3 appendices on: 1) challenges and opportunities in an OA model 2) five key priorities for action, and 3) stakeholder engagement questions. | All three phases support development of a model to facilitate prevention, early diagnosis, and access to evidence-based care for OA that that reflects the needs of the individual appropriate to the severity of their disease (pg.7).  In phase 1 the purpose was to identify the status of the management of osteoarthritis across Canada through a stakeholder engagement (pg. 7). In phase 2, the purpose was to identify evidence-based strategies and develop action plans to support osteoarthritis prevention, diagnosis, and management through a meeting (pg.10). In phase 3, the purpose was to identify evidence-based osteoarthritis strategies and assist in the implementation of those strategies (pg.10). | People with OA, The Arthritis Society, university departments, primary health care providers, health and wellness sector, professional associations/organizations, government/policy makers, community providers, private sector and industry, insurers, pharmacies, Arthritis Alliance Canada, media/marketing (pg.14). | Recommendations were developed through stakeholder participation in stakeholder engagement sessions in 2013 and a meeting hosted in May 2014.  Trans-Canada stakeholder engagement involved semi-structured interviews with researchers, practicing clinicians and policy makers (pg.7).  The meeting was developed and guided by a steering committee. The meeting took place in May 2014 and had 51 attendees, including researchers, clinicians, administrative and decision makers/policy makers and a range of stakeholders who represent professional associations and provide services for people with arthritis, including individuals from the health and wellness sector. The meeting consisted of presentations, with a person living with osteoarthritis as the keynote speaker, and breakout sessions (pg.9,10) |
| A Strategy to Reduce Hip and Knee Joint Replacement Surgery Wait Times  in Newfoundland and Labrador  Government of Newfoundland and Labrador  Department of Health and Community Services  2012 [38]  Arthritis; wait times | 16-page report containing: 1) introduction, 2) national wait time benchmarks, 3) provincial context, 4) wait time issues, 5) an overview of the strategy, 6) provincial numbers, 7) details on 5 goals including action items, and 8) conclusion. The document also includes an appendix on 1) Provincial Wait Times Data and 2) a figure demonstrating the percentage of patients receiving care within benchmarks by province in 2010. | A five-year Strategy designed to reduce wait times in the province for hip and knee joint replacement surgeries and ensure patients have access to hip and knee joint replacement surgeries within the national benchmarks (pg.4). | The Department of Health and Community Services, Provincial Government, Regional Health Authorities, healthcare professionals | To inform the development of this Strategy, the Department of Health and Community Services engaged the Centre for Research Healthcare Engineering from Ontario to perform a current state assessment of the Orthopedic wait lists in Eastern, Central and Western Health (pg.3).  To develop the Provincial Government’s Strategy to Reduce Hip and Knee Joint Replacement Wait Times, the Department of Health and Community Services consulted with senior staff in the three regional health authorities, orthopedic surgeons and family physicians in the Eastern, Central and Western Health regions (pg.5).  Goals are consistent with the 2011-2014 Strategic Plan of the Department of Health and Community Services, under the issues of quality and safety and improved access and increased efficiency (pg.5). |
| Self-management  support for Canadians  with chronic  health conditions: A focus for primary  health care  Health Council of Canada  2012 [39]  Arthritis; self-management | 60-page report containing: executive summary and five sections including: 1) introduction, 2) what helps patients succeed in self-managing, 3) how primary care providers can help patients succeed, 4) how policymakers can help patients and primary health care providers succeed, and 5) internet-based self-management supports, and a section for conclusions and recommendations. The document also contains four appendices on: 1) resources, 2) examples of self-management programs, 3) examples of self-management support initiatives, and 4) examples of government self-management strategies, frameworks, and plans. | Highlights success factors, barriers, innovative practices, opportunities, and resources to advance the delivery of self-management support, through better integration with primary health care and community-based services and through continued research in key areas (pg.4). | Primarily patients and families affected by chronic diseases, primary health care providers, and health system managers and policymakers across Canada  Also, peers, disease associations, community organizations and volunteer groups, and private sector companies (pg.8). | Not reported |
| Joint Action on Arthritis A Framework to Improve Arthritis Prevention and Care in Canada  Arthritis Alliance of Canada  2012 [40]  Arthritis; osteoarthritis | 49-page report containing: executive summary, introduction, and 3 sections on: 1) why arthritis must be addressed, 2) the Framework’s objectives and strategies, and 3) initial priorities for implementation. The document also contains 4 appendices on: 1) summary of supporting evidence, 2) links to related reports, 3) standards for arthritis prevention and care, and 4) references. | Engage stakeholders in identifying and planning how to implement strategies to improve prevention, detection, and management of arthritis (pg.7) through 1) communicating advancing knowledge and awareness of arthritis, 2) stimulating action around long-term strategies to improve prevention, and improve access to and delivery of arthritis care, and 3) supporting and broadening collaboration among governments and arthritis stakeholders in awareness, models of care and research (pg.2).    Serves as a mechanism to implement the *2005 Summit on Standards for Arthritis Prevention and Care* (pg.8) | Primarily governments and arthritis stakeholder organizations  Also people living with arthritis, educators, employers, insurers, health care providers, researchers (pg.7). | Summarizes recommendations gathered from January to September 2012, when over 100 stakeholders from 60 different organizations across Canada participated in workshops to provide expert advice. Stakeholder advice was used as supporting evidence in the development of strategies (pg.7).  Builds on the following past reports:   1. *2005 Summit on Standards for Arthritis Prevention and Care,* which was developed through collaboration among arthritis stakeholder organizations. 2. *2011 Canadian Arthritis Funding Landscape Review*, which was developed through a collaborative effort among Canada’s leading arthritis organizations to understand the current arthritis funding landscape in research, share their perspectives, and to develop recommendations for Arthritis funders. 3. *Impact of Arthritis in Canada: Today and Over the Next 30 Years 2011*, which was developed over a 3-year timeline of 3 workshops which brought together different key stakeholders for feedback and advice on improving care and reducing arthritis burden. A final report was developed and then reviewed by expert advisors. |
| The Impact of  Arthritis in Canada:  Today and Over The  Next 30 Years  Arthritis Alliance of Canada  2011 [41]  Arthritis | 51-page report containing: 1) executive summary, 2) introduction, 3) the approach, 4) simulations of the Canadian population, 5) details about the status and future of osteoarthritis, 6) details about the status and future of Rheumatoid Arthritis, and 7) conclusions and next steps. Also includes references, glossary, and an appendix on standards for arthritis prevention and care. | Better understand the burden of arthritis on Canadians living with the disease today and over the next 30 years and to investigate the potential impact of 4 targeted arthritis interventions and strategies to mitigate this burden (pg.12). Report also provides an outline of a comprehensive National Framework for Arthritis (pg.14). | Arthritis community, governments, broader health community (pg.14). | Uses clinical, survey and administrative data from cohorts of patients living with a diagnosis of OA or RA that has been confirmed by a physician to better understand the current state of arthritis in Canada. Information collected directly from patients is cross-linked with data from their healthcare providers and from administrative databases (e.g. billing databases).  The process leading to this report consisted of three workshops hosted by the Arthritis Alliance of Canada, including 1) expert workshop to identify data sources, 2) national arthritis alliance workshop to identify key intervention, and 3) national arthritis alliance workshop to review life at risk modeling results to build a final report (pg.22). |
| Improving Health Together: Policy Framework for Chronic Disease Prevention and Management  Government of Newfoundland and Labrador Department of Health and Community Services  2011 [42]  Arthritis; chronic disease prevention | 20-page report containing: 1) executive summary, 2) introduction, 3) overview of chronic disease in newfoundland and Labrador, 4) working together and building success (improving health together), 5) setting directions, which includes six policy statements, and a 6) conclusion. The document also includes an appendix demonstrating the expanded chronic care model. | Outlines a long-term approach for addressing chronic disease prevention and management in Newfoundland and Labrador. Lays the foundation for actions to be developed and implemented over several years (pg.3). | Government of Newfoundland and Labrador, people with arthritis. Also, community groups, employers, health care providers, and governments (pg.6). | Builds on other Provincial Government initiatives that seek to improve health and well-being such as the Provincial Wellness Plan which focuses on promoting and supporting healthy eating, physical activity and being smoke free (pg.8).  Uses the Expanded Chronic Care Model as a basis for policies and programs related to chronic disease prevention and management (pg.8). |
| Arthritis isn’t a big deal…  …until you get it.  Ask 4 million Canadians.  Report from the  Summit on Standards for  Arthritis Prevention and Care  Arthritis Alliance of Canada    2006 [43]  Arthritis; osteoarthritis; rheumatoid arthritis | 69-page report containing: 1) executive summary, 2) background, 3) key issues, including arthritis awareness,  prevention, and management, 4) nine topics for standards development, 5) a section detailing the summit on standards for arthritis prevention and care in November 1-3, 2005, 6) the standards for arthritis prevention and care, 7) action plan for standards implementation, 8) outstanding research questions, and 9) next steps. report also includes 4 appendices on 1) supporting evidence, 2) participants and teams, 3) support, and 4) agenda - the summit on standards for arthritis prevention and care November 1-3, 2005, and a section for references. | Outlines the Standards for Arthritis Prevention and Care, including inflammatory and osteoarthritis. The standards identify strategies within the context of awareness, prevention and management (pg.III) | People with arthritis, government, health care providers, health researchers, policy makers and industry (pg.2). | Standards built on earlier Supporting Evidence and The Arthritis Bill of Rights. A Planning Committee was created with broad input from all stakeholders. The Summit Planning Committee brought together representatives from across the broad arthritis community, the majority of which represented people living with arthritis of differing ages, genders, races/ethnicities, and geographic locations (pg.2).  Standards developed from Summit that involved two hundred Summit delegates who met over two days, November 1-2, 2005. On the first day, the standards from the nine topics were reviewed and modified during two sessions attended by approximately 20 delegates. On the second day, the potential barriers to, and facilitators of, standard implementation were similarly discussed twice (pg.13).  On January 24th, 2006, Arthritis Alliance of Canada’s Steering Committee met in Toronto to determine “next steps” for implementation of the standards. Each standard was reviewed and discussed with respect to its importance to people living with arthritis and three identified as requiring immediate attention (pg.35). |
| Arthritis and related conditions in Ontario  ICES research atlas    Institute for Clinical Evaluative Sciences (ICES) Toronto    2004 [44]  Arthritis | 146-page report containing: 1) authors affiliations, 2) acknowledgements, 3) about the organizations involved, 4) exhibits and appendices directory, 5) map guide on population geography and mapping boundaries, 6) overview, and 7) key findings and policy options. Also includes 7 chapters on 1) emerging issues, 2) burden of disease, 3) availability of services, 4) primary and specialist care, 5) use of medication, 6) surgical services, and 7) rehabilitation for total joint replacement | Provides an overview of the impact of arthritis and related conditions across Ontario, identifies strategies that may reduce the adverse consequences associated with arthritis, and provides a basis for initiatives to enhance access to care and services (pg.xv). | Policymakers, decisionmakers, health care professionals, the public, particularly individuals with arthritis (pg.xv). | Produced in partnership with the Arthritis Community Research and Evaluation Unit and The Arthritis Society, Ontario division (pg.xv).  Data obtained from provincial population health surveys, the Ontario Health Insurance Plan database, the Ontario Drug Benefits database, home care data, and databases on hospital admissions and day surgery procedures (pg.xv). |
| Making Arthritis Care in BC the Best in Canada    Arthritis Consumer Experts, Arthritis Research Centre of Canada, Canadian Arthritis Patient Alliance  Not reported [45]  Arthritis | 6-page document containing: 1) introduction 2) 12 recommendations related to arthritis. | Lists recommendations developed by British Columbia’s arthritis community to support arthritis prevention and care (pg.1). | Government of British Columbia (pg.1). | Not reported |
