# Supplementary material for: Policies in Canada fail to address disparities in access to person-centred osteoarthritis care: a content analysis
Source: BMC Health Serv Res. 2024 Apr 25;24:522. doi: 10.1186/s12913-024-10966-5 (PMC11044343; doi:10.1186/s12913-024-10966-5)
Supplement: Supplementary file 4 — Supplementary Material 4 [file 12913_2024_10966_MOESM4_ESM.docx]

**Additional File 4. Data extracted from included policies on person-centred care**

| Policy  Year | **Person-centred care domains** | | | | | |
| --- | --- | --- | --- | --- | --- | --- |
|  | Foster healing relationship  (Extend friendly greeting, make eye contact, speak in respectful manner, avoid judgmental attitude) | Exchange information  (Listen to concerns, prompt for additional details, understand needs, goals, circumstances and preferences, use lay language, ensure privacy) | Respond to emotions  (Actively inquire about feelings, acknowledge concerns, express empathy, note that such feelings are normal or common, suggest strategies to cope or mitigate emotions) | Manage uncertainty  (Offer rationale for tests or treatment, describe likelihood of risks and benefits using words, statistics or pictures) | Share decisions  (Describe treatment or management options, assess interest in shared decisions, provide information to enable shared decisions, suggest factors to consider in making decisions) | Enable self-management  (Set expectations for follow-up care, offer advice on self-care, provide take-home information, refer to other sources of information or support) |
| Arthritis Society, 2021 [29] | -- | Despite the prevalence of arthritis, the Working Group called out the frequency with which OA is discussed in dismissive terms (e.g., “it’s just age,” or “it’s just wear and tear”). Even the use of the term “elective” can be rather dismissive and should instead always be called “scheduled.” This impacts the timely utilization of publicly available disease prevention resources (e.g., obesity, physical inactivity, knee injury prevention), early diagnosis and treatment interventions in primary care/ interdisciplinary care, and investment in research to slow progression of OA (pg.5).  Don’t use language like ‘nonsurgical’ as it is extremely dismissive when somebody thinks they’re going to see a surgeon and then they’re told ‘you’re non-surgical’. Some of my patients think that means they will never receive surgery, and yet, three years later when they require surgery, they are left confused (pg.5). | -- | -- | -- | -- |
| Alberta Health Services, 2020 [30] | -- | -- | Addressing individual mental and psychological characteristics are important [for self-management]: What are the best ways to self-motivate? How do acute injuries and long-term conditions affect mood? How do bone and joint health practitioners collaborate with their mental health colleagues to increasingly provide a whole person, whole health approach? (pg.12). | -- | Patients will be engaged in collaborative and shared decision making, and will be partners in their care (pg.19). | -- |
| Bone and Joint Canada, 2019 [31] | -- | -- | -- | -- | -- | Promote and reinforce physical activity, including daily weight bearing, as a critical component of self-management through interactions with primary care and with the community sector (pg.4).  Participation in physical activity consistent with national guidelines needs to be encouraged through their primary care provider (pg.9).  Examples to improve diagnosis include physiotherapy in primary care and the use of Advanced Practice roles to support patient management prior to consideration for surgery. For treatment, individuals  should have access to education and therapeutic exercise (pg.11).  Facilitating the assessment of physical activity at regular intervals by the primary care provider including recommendations for changes in activity levels that reinforce positive behavior; education and linkages to appropriate local programs (pg.13). |
| Alberta Bone and Joint Health Institute, 2019 [32] | -- | -- | -- | -- | -- | -- |
| Health Quality Ontario, 2018 [33-35] | -- | Health care professionals should talk to patients about how osteoarthritis affects energy, mood, sleep, work, hobbies, family, and social life (19 pg.14)  Assess osteoarthritis-related symptoms using valid and reliable measures to understand a patient’s pain experience (19 pg.35) | -- | -- | Patients should receive a comprehensive assessment of needs (including social and psychological factors that may impact quality of life, and medical and emotional needs, preferences, and goals) within 3 months of diagnosis to inform their care plan. Care plans should be revised at every new symptom or goal (19 pg.14)  For people with symptomatic osteoarthritis in whom nonpharmacological treatments are insufficient to control symptoms, offer pain-relieving medication options in collaboration with the patient. Pain-relieving medications include treatments for associated mood or sleep disorders. With respect to medication, the discussion should include information about its benefits, when to take it, how much to take, how long to take it for, any possible side effects, and an agreement to reassess the response to treatment on a regular basis (19 pg.38)  Referral process should involve an integrated approach where there is collaboration, communication, and shared decision-making promote patient-centred care (19 pg.41). | People with osteoarthritis require support to develop an individualized, goal-oriented self-management plan; instruction on how to access resources when needed; and information on community-based services, programs, and online supports (33 pg.3).  Provide patients with referrals to different services and programs according to their needs (19 pg.5)  The sharing of information is an integral part of osteoarthritis management and should include family and caregivers, if appropriate. Information should be provided based on patient’s needs, their perception of their condition, their learning abilities, and their readiness to change (19 p.16)  Provide education (or refer to community-based education programs) in response to the needs of people with osteoarthritis to enhance their understanding of the condition and its management. Information should include all aspects of management and be reinforced and expanded upon at subsequent visits. Share information with family and caregivers, if appropriate (19 pg.17)  Provide patients with information about how to access local services, such as exercise classes, weight-management programs, and support groups (19 pg.20)  Work with people with osteoarthritis to support the development of an individualized, goal-oriented self-management plan that gives the person information and advice on the ongoing management of their symptoms and directs them to resources and other supports they may need. [Plans] should include information about how to access local services, such as exercise classes, weight-management programs, and support groups. [Plans] will also need to consider any other medical conditions you have that may impact your goals and abilities. Depending on [the patients] needs, [plans] might also include information about aids and devices such as suitable shoes, leg braces, orthotics, and hand grips. These things can help you stay active and function well (19 p.20).  Consider medical conditions that may affect goals and abilities in the development of self-management plans and adjust plans as condition and needs change (19 pg.21).  For your patients with osteoarthritis of the hip and knee, provide them with progressive neuromuscular training, muscle strengthening, and aerobic exercise of sufficient frequency, intensity, and duration to maintain or improve joint health and physical fitness. This may include referral to a supervised individual or group education and therapeutic exercise program. The program should address the person’s individual needs, circumstances, and self-motivation as identified in the clinical assessment. The program needs to be individually progressed; however, it can be provided in a group setting, depending on availability of local programs or facilities (19 pg.24)  Encourage patients to be physically active and provide patients with a plan including education and supports on the importance of physical activity to manage symptoms (19 p.26)  Work with patients to develop a plan to reach physical activity-related goals and refer to community programs that can help patients achieve their goals (19 pg.29)  Offer people who are overweight or obese weight-management strategies or refer to community programs for symptom relief (19 pg.31)  Provide patients with information and support to develop individual weight loss goals, learn problem-solving techniques to reach their goals, and receive follow-up visits to re-evaluate and discuss their goals (19 pg.32)  When clinically indicated, refer people with osteoarthritis for assessment and/or treatment by a health care professional with additional skills in osteoarthritis management. Primary care providers: Provide a detailed referral that includes the clinical assessment, comprehensive assessment results, the patient’s individualized care plan and self-management plan, and the clinical indication for referral. Tell your patient how they will be contacted about the referral appointment. Health care professionals with additional skills in osteoarthritis management: Communicate with the patient’s primary care provider to inform them of the timing of the referral response. After consultation, communicate the recommended plan for treatment and follow-up (if needed) to the patient and their primary care provider (19 pg.42). |
| Bone and Joint Canada, 2014-2015 [36,37] | -- | -- | -- | *--* | -- | People with OA need access to the most appropriate health care professional to ensure access to the best evidence-based care. This care includes education about their symptoms so that they can manage them effectively (35 pg.9).  Critical need to engage, educate and empower patients through providing researched-based information and road maps for patients so they understand how to participate in their own management (35 p.22). |
| Government of Newfoundland and Labrador  Department of Health and Community Services, 2012 [38] | -- | -- | -- | -- | -- | -- |
| Health Council of Canada, 2012 [39] | -- | -- | Tackle the social and emotional issues that often accompany  chronic disease through referral to a counsellor (pg.24). | -- | Underpinning self-management support is shared decision-making between patients and health care providers. Health care providers are to provide patients with complex information in clear and understandable terms and, when needed, to help patients develop skills in making decisions that support their physical and mental health. For example, patients should know and be empowered to ask, whenever a new medication is introduced, “How will this new drug interact with my other medications?” (pg.21). | Assessment tools are available to help patients and their health care providers understand where they are in terms of readiness to change. Health coaching by trained providers or peers can help patients move towards readiness (pg.16).  For a primary health care provider...by primary health care providers, we mean not only family doctors in solo or group practice, but also nurse practitioner-led clinics, doctors working with nurses in a primary health care practice, and interprofessional teams involving doctors, nurses, social workers, pharmacists, dietitians, and others. They may also include health care providers in disease-specific education clinics, and lay community health workers who may deliver chronic disease education and self-management support within their own ethnic, cultural, or geographic communities, ideally as part of a primary health care team…the goal of self management support is an informed and empowered patient with the skills and confidence necessary to manage his or her chronic conditions (pg. 21).  Providers and patients should collaboratively define problems and then develop realistic goals and a personalized action plan (pg.22).  Conduct a brief targeted assessment, including clinical severity and functional status, exploring the patient’s problems and goals, current self-management behaviours, readiness to change, and barriers to self-management (pg.22).  Provide disease-specific education and technical skills to teach patients to care for their specific conditions (e.g., how to manage multiple medications and avoid adverse interactions) and promote skills development (pg.22).  Providers and patients should collaboratively define problems and then develop realistic goals and a personalized action plan (pg.22).  Encourage the development of problem-solving skills to overcome barriers to change (pg.22).  Provide active follow-up and links to evidence-based community programs (pg.22).  Pursue multi-faceted interventions and provide comprehensive self-management support through an interprofessional team approach (pg.23).  Provide self-management support by using behaviour-change strategies as part of a regular clinical visit…the 5As is a set of well-tested behaviour-change techniques that health care providers can use to help patients develop realistic action plans and take other steps towards self-management. By incorporating multiple types of self-management support, the strategy becomes greater than the sum of its parts and is more likely to lead to better outcomes for patients. The Flinders Program for Chronic Care Management, developed in Australia, gives health care providers a care planning approach and tools to support the assessment, planning, and motivation of patients with chronic physical or mental health conditions and co-morbidities. The program includes a number of tools for assessment, interviewing, and goal setting designed to help providers and patients collaboratively identify issues, formulate a care plan, and monitor and review progress. Motivational interviewing (MI) is a patient-centred method of communication that helps patients explore and resolve their ambivalence about behavioural change and, as a result, become more motivated to change their health-related behaviours… Guided self-determination (GSD) is a structured coaching strategy designed for nurses to help patients with diabetes who struggle with blood sugar control. Designed for one-to-one and group settings, the method uses a series of worksheets and coaching techniques to address barriers to effective problem solving and to guide patients in setting goals… Guided care is a multi-faceted case management approach for patients with more complex chronic conditions. It involves a nurse working with a group of primary health care physicians as well as with patients and their caregivers. Through these collaborations, the nurse develops a care guide (for providers) and an action plan (for the patient/caregiver). The nurse also coordinates the roles of the various care providers involved, uses motivational interviewing techniques to coach the patient for self-management, provides a referral to a group self-management education program, smoothes the patient’s transitions between sites of care, and provides education and support for family caregivers (pg.25-26). |
| Arthritis Alliance of Canada, 2012 [40] | -- | Health care professionals recognize osteoarthritis as a significant health issue and treat it consistent with current best practice (p.24). | -- | -- | Incorporate patient preferences, including risk-benefit trade-offs into decision-making and prescribing of arthritis medications (pg.24). | Provide every patient with timely and equitable access to effective and appropriate self-management medical and surgical therapies (pg.24)  Enable self-management by providing on-line and hard copy educational materials for individuals living with arthritis and health care providers (pg.24). |
| Arthritis Alliance of Canada, 2011 [41] | -- | -- | -- | -- | -- | -- |
| Government of Newfoundland and Labrador Department of Health and Community Services, 2011 [42] | -- | -- | -- | -- | Individuals are treated with dignity and respect, and are valued in the decision-making processes regarding their health. All aspects of an individual’s life are considered in relation to their health including his or her social, economic, environmental, and physical needs. The stories, opinions, and concerns of individuals and key stakeholders are considered in the actions for the prevention and management of chronic disease (pg.9).  Most importantly, it is the individual who decides, with the support of a health care provider, if and when they want to be more involved and are able to develop skills and confidence to manage their health (pg. 11). | Individuals must receive clear, consistent, and accurate information about their health and health care services to support them in leading healthier lives. In addition, individuals must have the skills to use the information to make informed decisions and take action for better health (pg.12).  Sharing basic practice guidelines with individuals at risk for or living with a chronic disease helps them to better manage their health. When individuals understand the reason for the approach to care and the outcomes that are expected, they are more likely to comply with medication regimes, have recommended tests and improve healthy behaviours to best manage their health(pg.14).  Appropriate learning opportunities, with support from the community and health care providers, can encourage individuals with any type of chronic disease to be more active in their health (pg.11).  Through [various self-management supports], individuals learn to problem solve, set personal health goals, choose information to make appropriate decisions about their health, communicate with their health care providers and make changes to improve their health (pg. 11).  Peer-led group programs in the community combined with ongoing counselling and support from health care providers have been the most effective ways to produce long-term benefits (pg.11).  Examples of initiatives that could support individuals to manage their health include: 1) community-based chronic disease self-management programs, 2) initiatives that reduce barriers to participation in self-management programs, 3) telehealth services to support self-management, 4) Models of practice for health care providers to support self-management, 5) self-management models in other formats (such as web-based or video conferencing), 6) self-management approaches that support unique populations, 7) self-management electronic portals for individuals and health care providers (pg.11).  Referrals to community programs by health care providers greatly increase the likelihood of clients to participate, and ultimately to learn new skills and new information that will help them to better manage their health (pg.16). |
| Arthritis Alliance of Canada, 2006 [43] | -- | Health care professionals must recognize osteoarthritis as a significant health issue and treat it according to current guidelines (pg.22). | -- | -- | Patient preferences including risk-benefit trade-offs, must be incorporated into regulatory decision-making and prescribing of arthritis medications (pg.24). | Promote “active” waiting rather than current passive waiting i.e., pre-surgery education, rehabilitation, etc. during waiting period (pg.25). |
| Institute for Clinical Evaluative Sciences (ICES) Toronto, 2004 [44] | -- | -- | -- | -- | -- | -- |
| Arthritis Consumer Experts, Arthritis Research Centre of Canada, Canadian Arthritis Patient Alliance, No date [45] | -- | **--** | -- | -- | -- | -- |
