# Supplementary material for: Policies in Canada fail to address disparities in access to person-centred osteoarthritis care: a content analysis
Source: BMC Health Serv Res. 2024 Apr 25;24:522. doi: 10.1186/s12913-024-10966-5 (PMC11044343; doi:10.1186/s12913-024-10966-5)
Supplement: Supplementary file 5 — Supplementary Material 5 [file 12913_2024_10966_MOESM5_ESM.docx]

**Additional File 5. Data extracted from included policies on equitable access to OA care**

| Policy  Year | Prevalence of OA by intersectional factors  (e.g. age, sex/gender, geographic location, socioeconomic status, etc.) | Barriers to OA care by intersectional factors  (e.g. age, sex/gender, geographic location, socioeconomic status, etc.) | Strategies needed or recommended  to improve equitable access to and quality of OA care for equity-seeking groups |
| --- | --- | --- | --- |
| Arthritis Society, 2021 [29] | -- | -- | PATIENT  --  CLINICIAN  --  SYSTEM  --- |
| Alberta Health Services, 2020 [30] | -- | -- | PATIENT  --  CLINICIAN  --  SYSTEM  -- |
| Bone and Joint Canada, 2019 [31] | -- | -- | PATIENT  Self-management advice or programs  The needs of remote communities are different and as such there are a number of initiatives that are being undertaken and evaluated in Alberta to identify opportunities to meet those needs including a walking program to facilitate physical activity at a local level (pg.9).  CLINICIAN  --  SYSTEM  --- |
| Alberta Bone and Joint Health Institute, 2019 [32] | -- | Knowing when to seek help with symptoms and an unwillingness to become a burden to the health care system or their families were commonly described barriers to care. For some marginalized populations, such as the homeless, these barriers were even greater and could appear almost insurmountable (pg.5).  One of the challenges for patients navigating conservative treatment is that most of the treatment modalities fall outside of the publicly funded health system. Community educational programs, lifestyle coaching, exercise programs, physiotherapy, massage therapy, and dietary consultations are mostly privately funded. This results in inequity for patients unable or unwilling to fund these treatments out of pocket or who lack adequate private insurance coverage. This situation also leads patients who are financially able to entertain treatment options that are not based on scientific and medical evidence…the result is a complex navigation challenge where patients are forced to become subject matter experts in managing their disease. They are often faced with the difficult choice of paying out of pocket for privately-funded treatments of uncertain benefit, suffering with untreated joint pain until the disease progresses to end-stage, or aggressively pursuing scarce public services, with little guidance or information to assist in their decision-making (pg.6). | PATIENT  --  CLINICIAN  --  SYSTEM  -- |
| Health Quality Ontario, 2018 [33-35] | [Osteoarthritis] is more common in middle to older age (prevalence is 35% in those aged 80 years and older), affects more women than men, and is associated with other chronic health conditions such as depression and high blood pressure (19 pg.3) | Patient factors such as age, sex, obesity, comorbidities, etc. should not be barriers to referral for joint surgery (19 pg.46)  Disparity in the availability of programs and services related to nonsurgical management of osteoarthritis across the province. Geographic barriers prevent people from managing their condition, such as difficulty travelling long distances to access existing programs and services (33 pg.3)  Financial barriers, such as a lack of extended health insurance coverage for programs and services, may prevent people with osteoarthritis from properly managing their condition. Most community-based services for osteoarthritis (e.g., physiotherapy, occupational therapy, weight-management programs) are not covered under Ontario’s provincial health care plan (33 pg.3)    Specific population groups, such as Indigenous peoples, newcomers, refugees, and the homeless, face barriers in access to care. Factors affecting this lack of access include a lack of programs and self-management resources in different languages, as well as culturally safe care. Some of these population groups are also disproportionately affected by poverty, social isolation, and precarious employment; this, in turn, may impact access to effective osteoarthritis care (33 pg.3) | PATIENT  Education (via community agencies, translated)  [Healthcare organizations/professionals] should work with community organizations to leverage expertise in languages spoken most frequently in a specific region, and translate existing educational resources on osteoarthritis into these languages (33 pg.12).  Education (tailored, multiple formats)  Patient education should be available in accessible formats (written, electronic) and should be tailored to specific needs of the population (19 pg.18)  Self-management advice or programs (tailored, culturally relevant)  People with osteoarthritis should be provided services that are respectful of their gender, sexual orientation, socioeconomic status, housing, age, background (including self-identified cultural, ethnic, and religious background), and disability. Equitable access to the health system also includes access to culturally safe care. Language, a basic tool for communication, is an essential part of safe care and needs to be considered throughout a person’s health care journey. For example, in predominantly English-speaking settings, services should be actively offered in French and other languages (19 pg.5)  Self-management advice or programs (tailored, culturally relevant)  Community health centres and [Indigenous] health access centres may be able to support the development of culturally informed programs and self-management resources (33 pg.3).  CLINICIAN  --  SYSTEM  Formal tools or framework to evaluate the equity of programs or policies in healthcare organizations  Health Equity Impact Assessments should be employed [in healthcare organizations] to reduce health disparities between population groups (33 pg.5) |
| Bone and Joint Canada, 2014-2015 [36,37] | According to the World Health Organization, 9.6% of men and 18.0% of women older than 60 years of age worldwide have symptomatic osteoarthritis (OA) (35 pg.4).  2 of 3 people with arthritis are under 65 years and 10% report symptomatic knee OA by age 60 (35 pg.4). | -- | PATIENT  --  CLINICIAN  --  SYSTEM  -- |
| Government of Newfoundland and Labrador  Department of Health and Community Services, 2012 [38] | -- | -- | PATIENT  --  CLINICIAN  --  SYSTEM  -- |
| Health Council of Canada, 2012 [39] | -- | While group programs, such as the Chronic Disease Self-Management Program, are well established and seem to work well for some people, other Canadians are falling through the cracks. The underserved may be patients with lower incomes, less education, or more complex disease, or who are unable (for a variety of reasons) to join a group program (pg.4).  Canadians who have chronic conditions and who are in fair-to-poor health are more likely than the general public to be poorer, older, less educated, and living in rural areas. Advice to join a gym or eat healthier food may be very challenging for these people to comply with (pg.9).  Many patients with limited health literacy have poor abilities to engage in self-management and are less likely to attend peer-led, self-management programs. Self-management programs and resources should be designed to take low health literacy into account (pg.14).  Numerous studies have identified difficulties in recruiting certain groups for chronic disease self-management programs (ethnic minorities, indigenous communities, rural residents, older people, and people with low income or lower education) and have raised concern that participation tends to drop off as the course progresses (pg.15). | PATIENT  Self-management advice or programs (tailored, culturally relevant)  Broaden and deepen efforts to reach more Canadians who can benefit from self-management supports. A one-size-fits-all approach will not work because of the diversity of social and personal circumstances and disease-related challenges that patients and caregivers bring to their self-management efforts, and because of the diversity of client groups and settings in which self-management support is delivered across Canada. More specifically: 1) Build on existing programs that have shown good outcomes, but also try new approaches, including one-to one supports and multi-faceted interventions for people with multiple chronic conditions. Multi-faceted approaches combine more advanced support such as case management with fundamental self-management support such as goal setting and action planning. 2) Make in-person group programs more accessible through cultural or low-literacy adaptations, or by delivering them in a wider range of settings such as workplaces and assisted-living residences. 3) Pursue opportunities to provide self-management support online, but don’t put all the eggs in the technology basket because that might risk leaving the most vulnerable patients behind. As online services continue to develop, research is needed to provide more evidence about their impact and ensure they are integrated with offline services. 4) Target caregivers as a distinct client group, in addition to patients themselves, in all approaches to self-management support. 5) Use the power of peers as an important component of self-management support initiatives (pg.46).  Self-management advice or programs (tailored, culturally relevant)  Target underserved populations (e.g., ethnic minorities, indigenous communities, rural residents, older people, and people with low income or lower education that tend to have lower participation in programs) through: working in partnership with community health workers, elders, or existing multicultural services to develop culturally appropriate program materials in terms of language and traditional beliefs about health; removing practical barriers to participation by offering phone-based interventions…and delivering programs online, but also considering that people with financial or literacy challenges may not have access to a web-enabled device or be comfortable using it (pg.15).  CLINICIAN  --  SYSTEM  Train and mobilize lay health leaders  Target underserved populations (e.g., ethnic minorities, indigenous communities, rural residents, older people, and people with low income or lower education that tend to have lower participation in programs) through…training lay health workers from underserved communities to deliver self-management support (pg.46). |
| Arthritis Alliance of Canada, 2012 [40] | While both men and women get arthritis, two thirds of those affected in Canada are women (pg.9)  Arthritis is also particularly prevalent among [Indigenous] people[s]. According to the Public Health Agency of Canada’s latest arthritis surveillance report, the prevalence estimate for First Nations adults living both on- and off-reserve and for Metis adults is 1.3–1.6 times higher than the national estimate in the Canadian adult population (pg.11) | Middle-aged and older adults with OA report that their condition has a particularly devastating impact on employment, community mobility, heavy housework, leisure activities, social activities and close relationships (pg.10).  For many, gaining access to the right care and the right provider is a challenge. This is particularly true for people living in rural and remote areas, especially [Indigenous] populations, where distance and transportation costs are additional barriers (pg.22).  Access to cost-effective therapies, including drug therapies is limited for some across Canada due to inconsistent drug formularies. While gains have been made, there remains inequities in access to hip and knee replacement surgeries (pg.23) | PATIENT  --  CLINICIAN  Education (tailored to vulnerable groups)  Across all health professions, strengthening training and continuing education in musculoskeletal disorders would effectively develop and maintain the professional knowledge and skills necessary to effectively identify, assess, monitor and care for individuals with arthritis. Government, professional colleges and regulatory agencies, and arthritis stakeholders must focus their efforts and collaborate on the following strategies: 3.1 Strengthen the undergraduate/professional entry-level curricula related to arthritis as part of chronic disease prevention and management for all health care providers. 3.2 Promote consumer/patient and other stakeholder participation in health professional education related to arthritis. 3.3 Incorporate arthritis-related curriculum into post-graduate and specialty programs that address the needs of vulnerable groups. 3.4 Develop and implement a strategy to incorporate arthritis-related information and new research knowledge into continuing health professional education (p.19).  SYSTEM  -- |
| Arthritis Alliance of Canada, 2011 [41] | Incidence [of osteoarthritis] is expected to be higher among women than men (average women:men ratio of 1.43:1 over the next 30 years) (pg.30).  Prevalence of OA is shown to be higher among women than men (average ratio women:men ratio = 1.46:1 over the next 30 years) (pg.31).  As the Canadian population ages, OA prevalence is expected to increase and be highest among those over the age of 70 years (Figure 8). In 2010, approximately 49% of seniors over the age of 70 years are expected to be living with symptomatic OA. By 2040, this number is expected to increase to 71%. (pg.31). | -- | PATIENT  --  CLINICIAN  --  SYSTEM  -- |
| Government of Newfoundland and Labrador Department of Health and Community Services, 2011 [42] | Chronic disease becomes more common as people get older. Low incomes, poor social supports, and unhealthy physical environments, also influence the development of chronic disease. These factors combined can contribute to a decreased quality of life for individuals (pg.2).  Chronic disease and risk factor rates tend to be even higher among [Indigenous] people (pg. 4). | Social, economic, and environmental conditions influence a person’s ability to maintain good health, prevent chronic disease and manage the complications of disease. All determinants of health must be considered to achieve optimal health (pg.6).  Barriers to program participation [can include] low literacy, disabilities, transportation costs, distances to services, and access to plain language health (pg.9). | PATIENT  Education (tailored, different formats)  Group appointments and clinics for individuals having the same disease may be beneficial for some; for others, internet-based modules for self-management may be more effective. Some individuals may prefer to communicate or schedule appointments with their health care provider by email or through other electronic formats. As well, telehealth solutions such as video conferencing are becoming more widely used. Programs and services can be provided in a variety of ways, in addition to the traditional methods, to best meet the needs of the individual (pg.13).  CLINICIAN  --  SYSTEM  -- |
| Arthritis Alliance of Canada, 2006 [43] | Arthritis is up to two-and-a-half times as common in the [Indigenous] community living off reserve (Public Health Agency of Canada, 2003) as in non-[Indigenous] Canadians. Overall, 27% of [Indigenous] people living off reserve have arthritis compared with 16% of the general Canadian population. However, arthritis receives little attention as a significant health issue within the [Indigenous] community (pg.I). | Barriers to physical activity include: lack of motivation to exercise; arthritis related fatigue; lack of sustainability; cost; lack of community accessibility; inappropriateness to age and disease status; lack of clarity regarding how best to deliver information about physical activity to parents, caregivers, children, and health providers; and lack of knowledge about appropriate physical activity among persons who deliver/prescribe physical activity and sports (pg.7).  Despite the establishment of a Common Drug Review process in Canada, there is unacceptable inequity in access to proven, cost-effective therapies for arthritis and osteoporosis for patients with similar diagnoses and severity of disease (pg.8).  Potential Barriers [to raising awareness of arthritis among Canadian include] 1) Cost 2) Technological barriers 3) Human resources (expertise, skill, capacity) 4) Target group very large and geographically widely dispersed 5) Varied languages, cultures 6) Lack of public appeal of “arthritis” 7) Pre-conceived myths, beliefs about arthritis, e.g. “only old people get arthritis” | PATIENT  --  CLINICIAN  --  SYSTEM  -- |
| Institute for Clinical Evaluative Sciences (ICES) Toronto, 2004 [44] | In 2000-01, two-thirds of people with arthritis were women and nearly 3 out of every 5 people with arthritis were younger than 65 years of age (pg.xvii).  Arthritis was more common in women than in men, at 21.4% vs. 13.2% respectively, with women making up almost two-thirds of people with arthritis (pg.17).  Likelihood of reporting arthritis rises with age and is higher in women (pg.22).  People with higher levels of education were less likely to report arthritis (pg.22).  Individuals of Asian and other origins were less likely to report arthritis, while North American [Indigenous]s living off-reserve were twice as likely to report arthritis (pg.22).  Reporting poorer health, not surprisingly, increased with advancing age. For men with arthritis, the proportion reporting poor health increased from 12% in the 15–34 years age group, to almost 44% in the 75 years and older age group, and for women with arthritis from 23% to 46% (pg.26).  Arthritis was reported more frequently by women, older people, and people with lower levels of education and lower incomes. These findings are consistent with other surveys, suggesting that people who have arthritis may have fewer resources to deal with the consequences of this condition. The higher prevalence of arthritis among women also raises questions of whether targeted initiatives are necessary to meet the needs of this sector of the population (pg.35).  Burden of arthritis increases with age and is higher in women than men. Also, some indication that the burden may be higher in the [Indigenous] population (pg.35).  High prevalence of arthritis among [Indigenous peoples] demonstrates importance of taking initiatives to improve the health of [Indigenous] populations and considering arthritis in the provision of appropriate health services (pg.35). | Inadequate availability of arthritis health professionals throughout  the province clearly results in reduced access to care for arthritis. Regional disparities persist in availability of rheumatological and orthopaedic services (pg.3).  Shortages and poor distribution of these professionals [primary care physicians, rheumatologists, orthopaedic surgeons, and physical and occupational therapists] are major barriers to service access for arthritis management, particularly in rural and remote areas of Ontario (pg.5).  Reducing the impact of arthritis in Ontario requires dealing with inequities in access and provision related to geography, gender, and socioeconomic status to ensure all citizens have similar opportunities for access (pg.6).  Women and people with less education and/or lower income were more likely to have potential unmet need for total joint replacement (pg.107).  In older groups the rates of arthroscopic procedures are similar for men and women, despite a higher prevalence of arthritis in women. In this context, the degree of under use of knee replacement surgery for women is more than 3 times than that for men (pg.121). | PATIENT  --  CLINICIAN  --  SYSTEM  -- |
| Arthritis Consumer Experts, Arthritis Research Centre of Canada, Canadian Arthritis Patient Alliance, No date [45] | -- | -- | PATIENT  --  CLINICIAN  --  SYSTEM  -- |
